# *In vitro* protein expression changes in RAW 264.7 cells and HUVECs treated with dialyzed coffee extract by immunoprecipitation high performance liquid chromatography

**DOI:** 10.1038/s41598-018-32014-z

**Published:** 2018-09-14

**Authors:** Cheol Soo Yoon, Min Keun Kim, Yeon Sook Kim, Suk Keun Lee

**Affiliations:** 1 Department of Oral Pathology, College of Dentistry, Gangneung-Wonju National University, and Institute of Oral Science, Gangneung, Korea; 2 Department of Oral and Maxillofacial Surgery, College of Dentistry, Gangneung-Wonju National University, and Institute of Oral Science, Gangneung, Korea; 3 Department of Dental Hygiene, College of Health Sciences, Cheongju University, Cheongju, Korea

## Abstract

RAW 264.7 cells and HUVECs were compared to evaluate the effects of dialyzed coffee extract (DCE) and artificial coffee (AC). Immunoprecipitation high performance liquid chromatography (IP-HPLC) showed DCE-2.5- (equivalent to 2.5 cups of coffee a day) and DCE-5-induced protein expression that was beneficial to human health, i.e., they led to significant increases in proliferation-, immunity-, cellular protection-, antioxidant signaling-, and osteogenesis-related proteins but decreases in inflammation-, NFkB signaling-, cellular apoptosis-, and oncogenic signaling-related proteins in RAW 264.7 cells, and slight decreases in angiogenesis-related proteins in HUVECs. These protein expression changes were less frequently observed for DCE-10 treatment, while AC treatment induced very different changes in protein expression. We suggest that the favorable cellular effects of DCE were derived from minor coffee elements that were absent in AC, and that the reduced effects of DCE-10 compared with those of DCE-2.5 or DCE-5 might have been caused by greater adverse reactions to caffeine and chlorogenic acid in DCE-10 than DCE-2.5 or DCE-5. IP-HPLC results suggested that minor coffee elements in DCE might play beneficial roles in the global protein expression of proliferation-, immunity-, anti-inflammation-, cell protection-, antioxidant-, anti-apoptosis-, anti-oncogenesis-, and osteogenesis-related proteins in RAW 264.7 cells and enhance anti-angiogenic signaling in HUVECs.

## Introduction

Coffee is a favorite drink worldwide, and many authors have investigated the effects of caffeine and chlorogenic acids (major components of coffee) in clinical and cell-based experiments. However, published results are controversial with respect to its effects on cardiovascular diseases, inflammation, diabetes, Parkinson disease, cancer, and other diseases^1–3^. In addition to caffeine and chlorogenic acids, many other minor coffee elements, such as, polyphenols, diterpenes (kahweol and cafestol), melanoidins, and trigonelline have also been identified and investigated^4–7^. Nevertheless, it is presumed that other coffee constituents may have pharmacological effects and act in synergistic or antagonistic manners.

The beneficial pharmacological effects of coffee mentioned in the literature include anti-inflammatory, anti-oxidant, anti-angiogenic, anticancer, chemoprotective, and hepatoprotective effects^8–11^. The anti-cancer effects of coffee has been observed in different cancer cells, including human lung adenocarcinoma A549 cells, hepatocellular carcinoma cells, and oral squamous carcinoma cell lines (HN22 and HSC4)^12–15^, and its anti-inflammatory, anti-oxidant, and anti-angiogenic effects have been reported in HUVECs, NIH3T3 cells, and lipopolysaccharide-activated RAW264.7 cells^16–18^. The present study was undertaken to examine changes in protein expression in macrophages, which can engulf coffee elements *in vitro*. We used non-stimulated RAW 264.7 cells derived from murine macrophages and cultured them in antigen-deactivated medium.

In the present study, we used dialyzed coffee extract (DCE), which can be absorbed by diffusion through mucosal epithelium in the gastro-intestinal tract. Preliminary studies on the bio-compatibility of DCE and RAW 264.7 cells showed no cytotoxic effect and little antigenic stimulation within a range of DCE doses^19,20^. Furthermore, mice injected intraperitoneally with DCE showed no histological changes in spleen or liver and remained healthy for more than six months.

Immunoprecipitation high performance liquid chromatography (IP-HPLC) is used to determine protein expression levels versus reference controls. Generally, IP-HPLC is comparable to ELISA; the former uses protein A/G agarose beads in buffer solution and UV spectroscopy to determine whole areas of protein peaks, and the latter uses fluorescence-conjugated antibody fixed in plastic wells and fluoroscopy to measure the highest intensity of fluorescence excitation^21,22^. In IP-HPLC procedures, the mixtures of protein sample and antibody-bound protein A/G agarose beads are incubated in a chaotic state by stirring, and after multiple washes of agarose beads, the target protein is eluted and analyzed by the automatic HPLC system using stable UV spectroscopy. Multiple trials have shown that IP-HPLC can detect protein expression changes accurately and reproducibly (±5% standard deviation). In the present study, IP-HPLC was used to assess the expression of different functional proteins (n = 189) in RAW 264.7 cells treated with DCE or AC (1 mM chlorogenic acid and 2 mM caffeine) *in vitro*.

## Results

### Cellular proliferation as assessed by counting after culture for 24 hours

**Figure 1:**
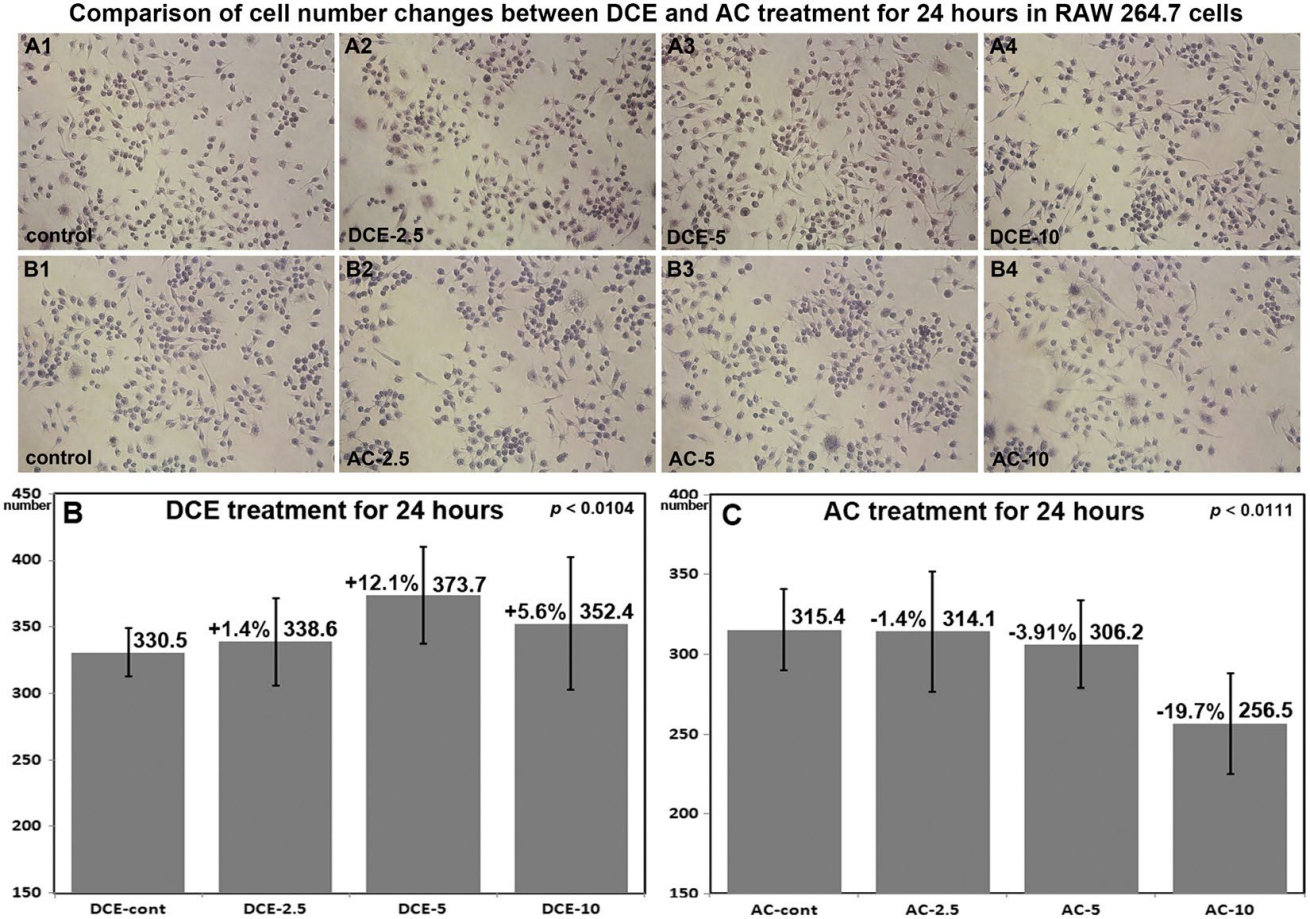
Comparison of cell number changes induced by DCE and AC in RAW 264.7 cells. Cultured cells on Petri-dishes were directly counted in multiple digital images (x200). The results are represented as mean ± STD, n = 24 to 35. The statistical difference was analyzed by Chi-squared test. The raw data for the cell counting analysis were shown in supplement 1.

RAW 264.7 cells were loosely and evenly distributed on the surfaces of Petri-dishes, treated with DCE-2.5, DCE-5, DCE-10, AC-2.5, AC-5, or AC-10 for 24 hours, fixed with neutral 10% formalin solution, and stained with hematoxylin. Cell numbers were determined by counting at x200 using an image analysis program (IMT i-solution ver 21.1, Vancouver, Canada). Numbers of RAW 264.7 cells were greater after treatment with DCE-2.5 or DCE-5 by 1.4% and 12.1%, respectively, versus controls, but reduced by 5.6% by DCE-10. By contrast, RAW cell numbers were reduced by AC-2.5, AC-5, and AC-10 to 1.4%, 3.9%, and 19.7%, respectively (Fig. 1B,C, Supplement 1). These results showed that DCE induced RAW 264.7 cell proliferation, whereas AC had the opposite effect, suggesting that these increases in cell numbers could reflect increases in antigen presenting cells and enhanced cellular immunity.

### Effects of DCE and AC on the expression of proliferation-related proteins in RAW 264.7 cells

**Figure 2:**
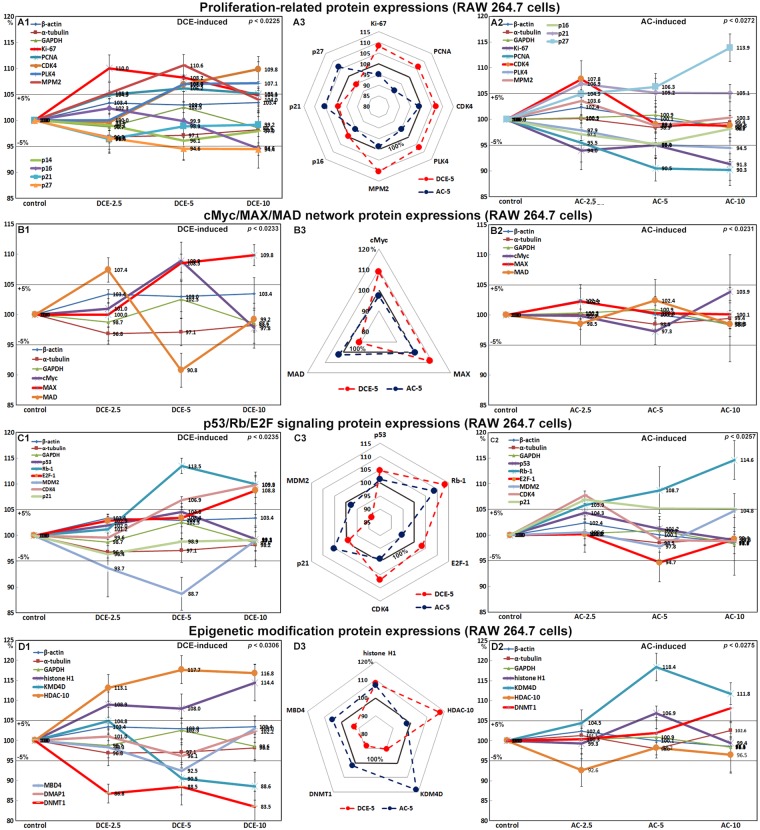
Expression levels of proliferation-related proteins (**A**), cMyc/MAX/MAD signaling proteins (**B**), p53/Rb/E2F signaling proteins (**C**), and epigenetic modification-related proteins (**D**) after DCE (A1,B1,C1,D1) or AC (A2,B2,C2,D2) treatment in RAW 264.7 cells as determined by IP-HPLC. A distinct contrast in protein expression between DCE-5 and AC-5 treatments was found in the radial graphs of (A3,B3,C3,D3).

RAW 264.7 cells treated with DCE-2.5 or DCE-5 showed significantly higher expressions of proliferation-activating proteins (Ki-67, 110%), proliferating cell nuclear antigen (PCNA, 106.2%), cyclin dependent kinase 4 (CDK4, 109.8%), PLK4 (107.1%), and mitotic protein monoclonal 2 (MPM2, 110.6%), and significantly lower expressions of proliferation-inhibiting proteins, p14 (96.1%), p16 (94.6%), p21 (96.4%), and p27 (94.4%), than non-treated controls, while cells treated with DCE-10 showed lower expressions of proliferation-activating proteins than cells treated with DCE-2.5 or DCE-5 (Fig. 2A1). The expression levels of p14 and p21 changed less then ±5% in response to DCE, similarly to the control housekeeping proteins (β-actin, α-tubulin, and glyceraldehyde-3-phosphate dehydrogenase, GAPDH). By contrast, RAW 264.7 cells treated with AC-2.5, AC-5, or AC-10 showed gradual reductions in the expression of proliferation-activating proteins (Fig. 2A2). These results suggested that DCE-2.5 and DCE-5, but not AC, enhanced the proliferation of RAW 264.7 cells.

### Effects of DCE and AC on the expression of cMyc/MAX/MAD signaling proteins in RAW 264.7 cells

RAW 264.7 cells treated with DCE-2.5 or DCE-5 showed slight increases in the expression of V-Myc myelocytomatosis viral oncogene homolog (cMyc, 108.9%) and Myc-associated factor X (MAX, 109.8%) but marked decreases in MAD (90.8%) expression versus non-treated controls, whereas cells treated with DCE-10 showed similar changes in the expression of MAX and MAD but marked reductions in cMyc (Fig. 2B1). By contrast, RAW 264.7 cells treated with AC-2.5 or AC-5 showed minimal changes in the expressions of cMyc, MAX, and MAD, whereas AC-10 induced a slight increase in cMyc expression (Fig. 2B2). These results suggested that DCE enhanced cMyc/MAX signaling in RAW 264.7 cells and thus induced proliferation.

### Effects of DCE and AC on the expression of p53/Rb/E2F signaling proteins in RAW 264.7 cells

RAW 264.7 cells treated with DCE-2.5 or DCE-5 showed increases in the expressions of p53 (104.6%), retinoblastoma-1 (Rb-1, 113.5%), and E2F-1 (103.4%) versus non-treated controls, and DCE-10 increased the expressions of Rb-1 (109.9%) and E2F-1 (108.8%). The slight increase in p53 expression induced by DCE-5 corresponded to a marked reduction in mouse double minute 2 homolog (MDM2) expression (Fig. 2C1). By contrast, RAW 264.7 cells treated with AC-2.5, AC-5, or AC-10 showed minimal changes in E2F expression, although AC-10 induced marked Rb-1 expression (Fig. 2C2). E2F expression was consistently enhanced by DCE but not by AC, which indicated that DCE-2.5 and DCE-5, on the one hand, and AC, on the hand, influenced p53/Rb/E2F signaling differently in RAW 264.7 cells. These results suggested that DCE positively activated p53/Rb/E2F signaling leading to RAW 264.7 cell proliferation concurrently with the increase in cMyc/Max and proliferation-related proteins.

### Effects of DCE and AC on the expression of epigenetic modification-related proteins in RAW 264.7 cells

RAW 264.7 cells treated with DCE-2.5, DCE-5, or DCE-10 showed marked increases in the expression of histone H1 (114.4%) but marked decreases in the expression of lysine-specific demethylase 4D (KDM4D, 88.6%), DNA (cytosine-5)-methyltransferase 1 (DNMT1, 83.5%), methyl-CpG binding domain 4 (MBD4, 92.5%), and DNA methyltransferase 1 associated protein 1 (DMAP1, 96.1%), followed by compensatory increase in histone deacetylase 10 (HDAC-10, 117.7%) (Fig. 2D1), while cells treated with AC showed minimal expression changes in histone H1and HDAC-10 but a marked increase in KDM4D (118.4%) (Fig. 2D2). These results suggested that DCE might enhance histone acetylation, reduce histone methylation, and activate DNA transcription in RAW 264.7 cells, while AC might induce histone methylation and inactivate DNA transcription. Therefore, DCE might participate in epigenetic modifications as well as cytoplasmic protein signaling.

### Effects of DCE and AC on the expression of translation-related proteins in RAW 264.7 cells

**Figure 3:**
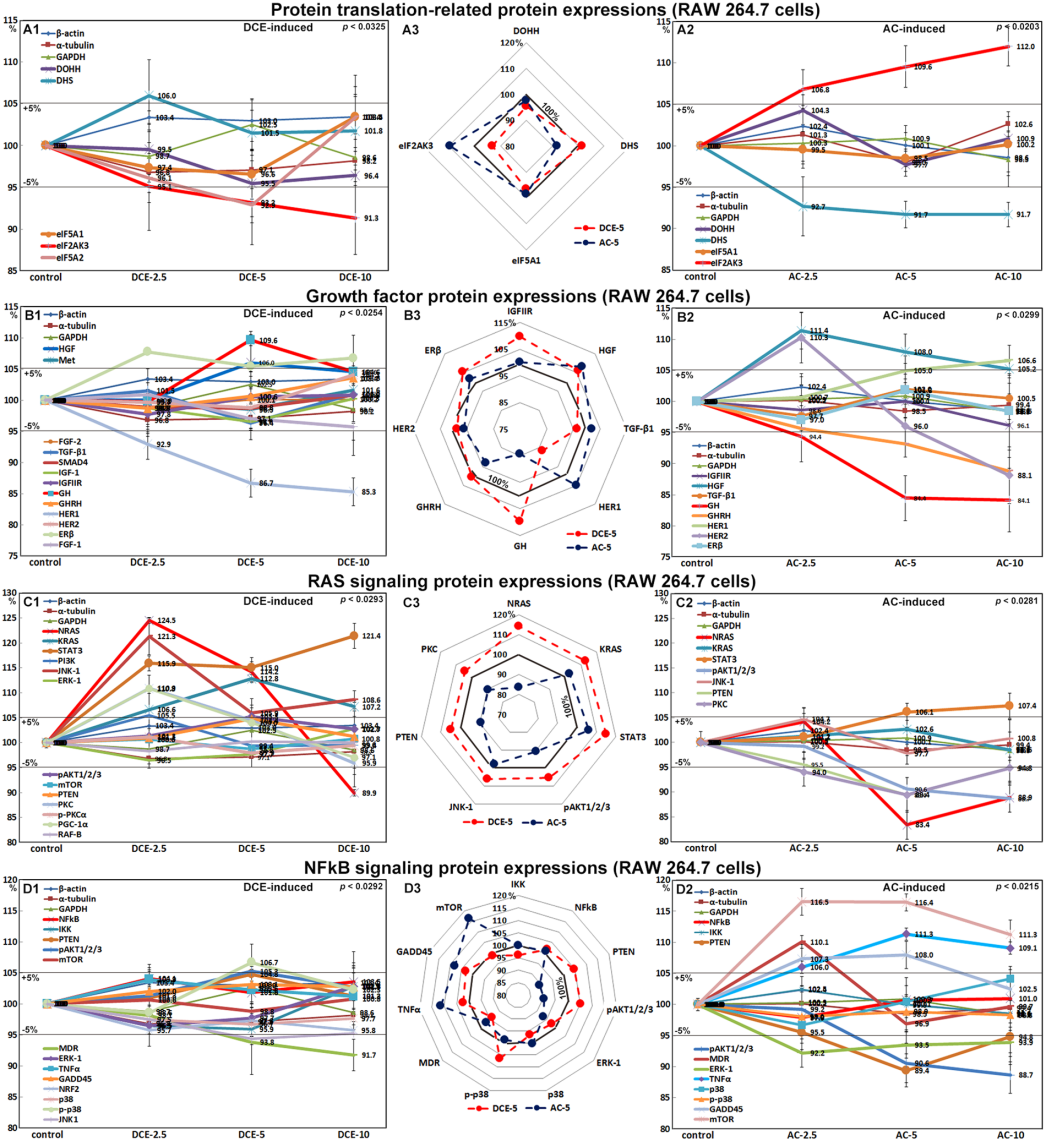
Expression of translation-related proteins (**A**), RAS signaling proteins (**B**), growth factor-related proteins (**C**) and NFkB signaling proteins (**D**) after DCE (A1,B1,C1,D1) or AC (A2,B2,C2,D2) treatment in RAW 264.7 cells as determined by IP-HPLC. A large contrast in protein expressions between DCE-5 and AC-5 treatments was observed in the radial graphs of (A3,B3,C3,D3).

RAW 264.7 cells treated with DCE-2.5, DCE-5, and DCE-10 showed slight increases in deoxyhypusine synthase (DHS) (106%) but slight decreases in eukaryotic translation initiation factor 5A1 (eIF5A1) and eIF5A2 protein levels (96.6% and 92.9%, respectively) compared with non-treated controls. However, the decreases in eIF5A1 and eIF5A2 were concurrent with the decrease in eIF2AK3 (91.3%), which could lead to a rapid reduction of translational initiation and repression of global protein synthesis (Fig. 3A1). Deoxyhypusine hydroxylase (DOHH) protein expression was also slightly decreased to 95.5% by DCE-5 and DCE-10, whereas AC slightly reduced DHS expression to 91.7% and had almost no effect on eIF5A-1 expression (98.5% to 100.2%) (Fig. 3A2). Because eIF5A-1 can function as a translation initiation and elongation factor depending on the presence of hypusination of its lysine residues by DHS and DOHH^23^, the increase in DHS expression after DCE-2.5 treatment might indicate the induction of protein translation by increasing eIF5A hypusination; however, these expression levels were reversed by AC treatment. These results suggest that DCE affects the basal protein translation level for cellular proliferation and different types of functional activation.

### Effects of DCE and AC on the expression of RAS signaling proteins in RAW 264.7 cells

DCE significantly affected the expression of RAS signaling proteins in RAW 264.7 cells. DCE-2.5 or DCE-5 markedly increased the expressions of neuroblastoma RAS viral oncogene homolog (NRAS, 124.5%), V-Ki-ras2 Kirsten rat sarcoma viral oncogene homolog (KRAS, 112.8%), signal transducer and activator of transcription-3 (STAT3, 115.9%), and pAKT1/2/3 (105.3%), but it slightly decreased the expression of extracellular signal-regulated protein kinases-1 (ERK-1, 96.5%). The expression levels of JNK-1 and protein kinase C (PKC) were markedly increased by DCE-2.5 to 121.3% and 110.9%, respectively, but progressively decreased after DCE-5 or DCE-10 treatment (Fig. 3B1). By contrast, RAW cells treated with AC-2.5, AC-5, or AC-10 showed gradual decreases in the expression of NRAS (83.4%), pAKT1/2/3 (88.7%), and PKC (89.4%), but slight increases in STAT3 (107.4%) (Fig. 3B2), indicating that DCE markedly enhanced RAS signaling to a greater extent in RAW 264.7 cells than AC.

### Effects of DCE and AC on the expression of growth factor-related proteins in RAW 264.7 cells

RAW 264.7 cells treated with DCE-2.5, and DCE-5 showed marked increases in the expression of growth hormone (GH, 19.6%) and growth hormone-releasing hormone (GHRH, 13.6%), slight increases in estrogen receptor beta (ERβ, 107.8%) expression, and marked reductions in human epidermal growth factor receptor 1 (HER1, 86.7%), but no effect on the expression of HER2 was observed (98.8–99.7%) (Fig. 3C1). RAW 264.7 cells treated with AC-2.5, AC-5 or AC-10 showed slight increases in the expression of hepatocyte growth factor (HGF, 108%), HER1 (106.6%), and HER2 (110.3% by AC-2.5), but a gradual decrease in GHRH (88.8%), GH (84.1%), and HER2 (88.1%) by AC-10. Alternately, the expression of general growth factors, that is, transforming growth factor- β1 (TGF-β1), IGFIIR, and ERβ, changed the minimum to less than ±5% similarly to the control housekeeping proteins (Fig. 3C2). These results suggested that the increased expression of GH, GHRH, and ERβ by DCE-2.5 or DCE-5 might be associated with enhanced RAS signaling and positively impact human health and that the marked reductions observed in the expression of HER1, HER2, and IGFIIR after DCE-2.5 or DCE-5 treatment might be useful for the treatment of different human diseases, such as breast cancer and diabetes.

### Effects of DCE and AC on the expression of NFkB signaling proteins in RAW 264.7 cells

RAW 264.7 cells treated with DCE showed gradual decreases in the expression of nuclear factor kappa-light-chain-enhancer of activated B cells (NFkB) signaling proteins. The expression of tumor necrosis factor-α (TNFα) and NFkB changed the minimum to less than ±5% similarly to the control housekeeping proteins after DCE-5 or DCE-10 treatment, whereas the expression levels of MDR and JNK1 were reduced to 91.7% and 94.4%, respectively. The mitogen-activated protein kinase p38 showed enhanced phosphorylation, and consequently p-p38 expression increased slightly to 106.7% after DCE-5 treatment (Fig. 3D1). RAW 264.7 cells treated with AC showed gradual increases in TNFα (111.3%) and MDR (110.1%) expression, which became marked after AC-5 treatment. However, the expression of NFkB and ikappaB kinase (IKK) was minimally changed. Cells treated with AC-2.5 showed a slight increase in MDR expression, and this increase was lower in cells treated with AC-5 or AC-10. By contrast, the expression of pAKT (88.7%), ERK-1 (92.2%), and phosphatidylinositol-3-kinases (PTEN, 89.4%) was slightly decreased after AC treatments (Fig. 3D2). These results suggested that in RAW 264.7 cells treated with DCE, NFkB signaling was not affected and the cells were in an unstressful condition similarly to the non-treated control, whereas treatment with AC increased NFkB signaling, increased the expression of TNFα and MDR, and decreased the expression of pAKT, ERK-1, and PTEN, indicating increased cellular stress.

### Effects of DCE and AC on the expression of immunity-related proteins in RAW 264.7 cells

**Figure 4:**
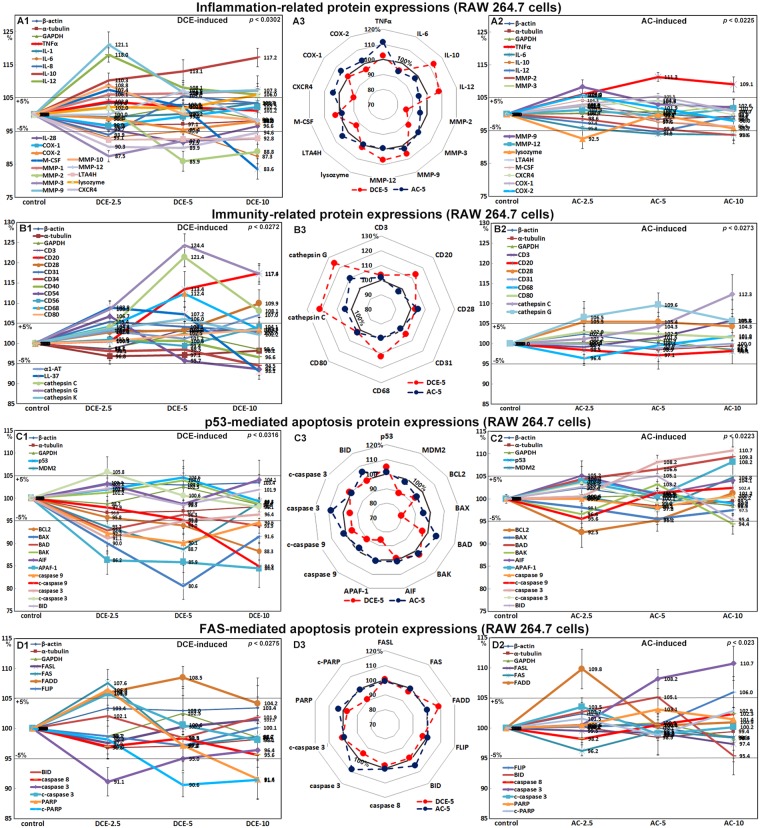
Expression of immunity-related proteins (**A**), inflammatory proteins (**B**), p53-mediated apoptosis-related proteins (**C**), and FAS-mediated apoptosis proteins (**D**) after DCE (A1,B1,C1,D1) or AC (A2,B2,C2,D2) treatment in RAW 264.7 cells as determined by IP-HPLC. A large contrast in protein expression between DCE-5 and AC-5 treatments was observed in the radial graphs of (A3,B3,C3,D3).

RAW 264.7 cells treated with DCE showed significant increases in the expression of cathepsin C (121.4%), cathepsin G (124.4%), cluster of differentiation 20 (CD20, 117.4%), CD28 (109.9%), CD31 (105.4%), and CD68 (112.4%). The expression levels of cathepsin G, cathepsin C, CD31, and CD68 were markedly increased by DCE-5, but less so by DCE-10. In particular, cathepsin G was overexpressed in cells treated with DCE-5 or DCE-10. The expression levels of CD20 and CD28 were elevated by all DCE treatments and were marked after treatment with DCE-10 (Fig. 4A1). By contrast, cells treated with AC showed less-significant changes in the expression of immunity-related proteins, although AC-5 and AC-10 slightly increased the expression of cathepsin G and cathepsin C to 109.6% and 112.3%, respectively (Fig. 4A2). These results indicated that DCE activated RAW 264.7 cells by increasing the expression of macrophage biomarkers, i.e., cathepsin G, cathepsin C, CD31, and CD68, and stimulated these cells by increasing the expression of the immunogenic proteins CD20, CD28, CD31, CD40, and CD68, thereby resulting in the cellular immunity activation of RAW 264.7 cells.

### Effects of DCE and AC on the expression of inflammation-related proteins in RAW 264.7 cells

RAW 264.7 cells treated with DCE showed a down-regulation of the matrix inflammatory factors interleukin-6 (87.3%) and IL-28 (91.5%), but marked up-regulation of the cellular inflammatory factors IL-1 (103.7%), IL-8 (105.6%), and IL-12 (118%). However, the anti-inflammatory factor IL-10 was markedly up-regulated to 117.2% after DCE-10 treatment. The expression levels of matrix metalloproteases (MMPs) after DCE treatment were variable; MMP-1, MMP-9, MMP-10, and MMP-12 were up-regulated to 107.3%, 121.1%, 108.8%, and 107.1% while MMP-2 and MMP-3 were markedly down-regulated to 85.9% and 87.5%, respectively. In particular, the expression levels of leukotriene-A4 hydrolase (LTA4H), C-X-C chemokine receptor type 4 (CXCR4), M-CSF, and cyclooxygenase 2 (COX2) were dramatically reduced to 92.4%, 89.9%, 83.6%, and 95.4% by DCE, respectively, but TNFα and COX1 showed a small change less than ±5% (Fig. 4B1). By contrast, RAW 264.7 cells treated with AC-5 or AC-10 showed marked TNFα up-regulation to 111.3%, and cells treated with AC-2.5 showed a slight increase in the expression of MMP-9 (108.3%), COX1 (105.5%), COX2 (105.8%). However, AC caused non-significant increases in the expression of inflammatory proteins (Fig. 4B2). These results suggest that DCE induced anti-inflammatory signaling in RAW 264.7 cells by down-regulating LTA4H, CXCR4, COX2, interleukins, and matrix metalloproteases and not affecting the expression of COX1, while these anti-inflammatory effects were not observed after AC treatment.

### Effects of DCE and AC on the expression of p53-mediated apoptosis-related proteins in RAW 264.7 cells

Treatment of RAW 264.7 cells with DCE-2.5 or DCE-5 did not up-regulate the expression of p53-mediated apoptosis-related proteins, other than a slight increase in p53 expression (104.6%) and a concurrent decrease in MDM2 expression (88.7%), but it markedly down-regulated p53-mediated apoptosis-related proteins, such as BCL2 associated death promoter (BAD, 92.9%), BCL2 antagonist/killer (BAK, 98.7%), BCL2-associated X (BAX, 80.6%), apoptosis-inducing factor (AIF, 98.7%), apoptotic protease-activating factor 1 (APAF-1, 84.4%), caspase 9 (90.1%), cleaved caspase 9 (c-caspase 9, 84.9%), caspase 3 (91.1%), and c-caspase 3 (98.1%). The expression levels of p53-mediated apoptosis-related proteins were consistently diminished by DCE-10 (Fig. 4C1). RAW 264.7 cells treated with AC-5 or AC-10 showed slight increases in the expression of BAD (109.3%), APAF-1 (108.2%), c-caspase 9 (102.4%), and c-caspase 3 (103.5%), suggesting a slight activation of p53-mediated apoptosis but otherwise no significant changes in the expression of p53-mediated apoptosis-related proteins (Fig. 4C2). These results suggested that DCE, but not AC, might significantly reduce p53-mediated apoptosis signaling in RAW 264.7 cells.

### Effects of DCE and AC on the expression of FAS-mediated apoptosis-related proteins in RAW 264.7 cells

RAW 264.7 cells treated with DCE-2.5 showed slight increases in the expression of FAS (107.6%) and FAS-associated via death domain (FADD, 106%), although cells treated with DCE-5 or DCE-10 showed a gradual decrease in the expression of FAS (97.1%) and FADD (104.2%). The expression levels of caspase 8 and caspase 3 were consistently down-regulated to 97% and 91.1% by DCE at all three concentrations, respectively. Additionally, the expression of FASL, FLIP, and BID showed a minimum change of less than ±5% similarly to the control housekeeping proteins (Fig. 4D1). Whereas RAW 264.7 cells treated with AC-2.5 showed a slight FADD up-regulation (109.8%), cells treated with AC-5 or AC-10 showed a slight up-regulation of caspase 3 (110.7%) and non-significant changes in c-caspase 3, respectively. Changes in the expression levels of other FAS-mediated apoptosis-related proteins by AC treatments were non-significant. The expression of poly-ADP ribose polymerase (PARP) was markedly increased to 106.4% by DCE-2.5 but reduced to 97.2% by DCE-5 and further reduced to 91.6% by DCE-10, whereas the expression of c-PARP was consistently reduced to 90.6% at all three DCE concentrations, which contrasted with the non-significant changes in PARP and cleaved PARP levels induced by AC at all three concentrations (Fig. 4D2). These results suggested that RAW 264.7 cells treated with DCE tended not to undergo FAS-mediated cellular apoptosis and that cells treated with AC-5 or AC-10 were more likely to undergo FAS-mediated cellular apoptosis.

### Effects of DCE and AC on the expression of antioxidant-related proteins in RAW 264.7 cells

**Figure 5:**
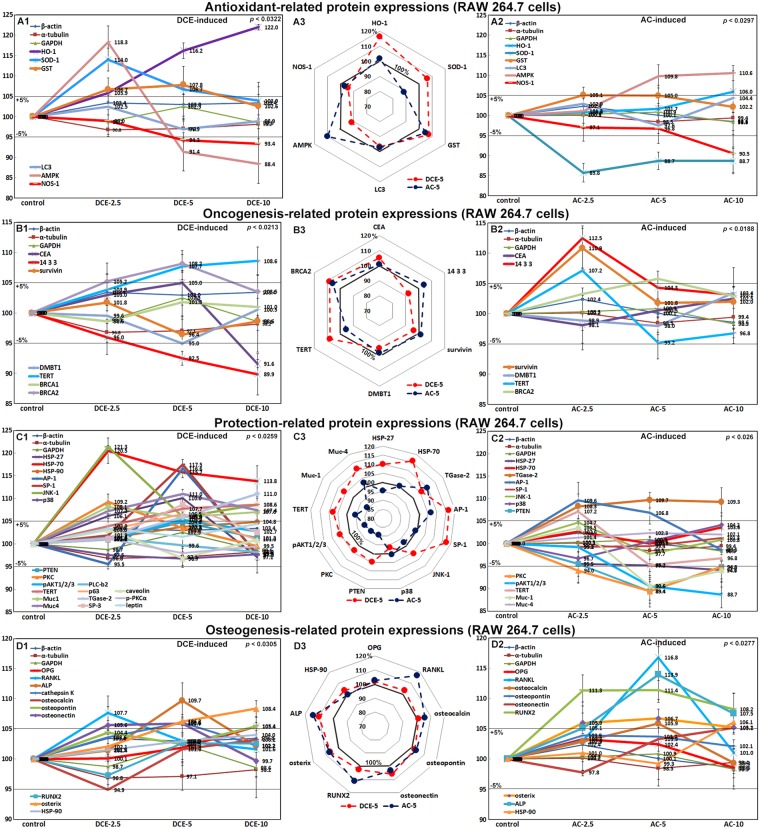
Expression of antioxidant-related proteins (**A**), oncogenic proteins (**B**), cell protection-related proteins (**C**), and osteogenesis-related proteins (**D**) after DCE (A1,B1,C1,D1) or AC (A2,B2,C2,D2) treatment in RAW 264.7 cells as determined by IP-HPLC. A large contrast in protein expression between DCE-5 and AC-5 treatments was observed in the radial graphs of (A3,B3,C3,D3).

RAW 264.7 cells treated with DCE-2.5 showed marked increases in AMP-activated protein kinase (AMPK, 118.3%), microtubule-associated protein 1 A/1B light chain 3 (LC3, 102.5%), and superoxide dismutase-1 (SOD-1, 114%), but the up-regulated expression of these proteins was reduced after treatment with DCE-5 or DCE-10 to 88.4%, 96.9%, and 103.9%, respectively. The expression of heme oxygenase 1 (HO-1) and glutathione S-transferase (GST) was gradually up-regulated to 122% and 107.8% by all three DCE concentrations, but more so after DCE-10 treatment, while the expression of NOS-1 was gradually down-regulated to 93.4% after DCE treatment (Fig. 5A1). Whereas RAW 264.7 cells treated with AC at all three concentrations showed marked decreases in SOD-1 (85.8%), AC-5 or AC-10 slightly increased AMPK expression to 110.6% (Fig. 5A2). These results indicated that DCE, but not AC, enhanced the activation of antioxidant effects of SOD-1, HO-1, GST, and LC3 in RAW 264.7 cells.

### Effects of DCE and AC on the expression of oncogenic proteins in RAW 264.7 cells

RAW 264.7 cells treated with DCE showed reduced expressions of oncogenic proteins, that is, deleted in malignant brain tumors 1 (DMBT1, 95%), 14-3-3 (89.9%), and survivin (96.4%), compared to non-treated controls. Cells treated with DCE-5 or DCE-10 showed a slight up-regulation of telomerase reverse transcriptase (TERT) to 108.6% (Fig. 5B1), whereas cells treated with AC-2.5 showed significant up-regulation of survivin (110.9%) and 14-3-3 (112.5%) and a down-regulation of TERT (95.2%) (Fig. 5B2). These findings suggested that DCE treatment did not up-regulate oncogenic protein expression in RAW264.7 cells but rather had a slight anti-oncogenic effect by down-regulating the expression of DMBT1, 14-3-3, and survivin. By contrast, AC consistently increased the expression of oncogenic proteins.

### Effects of DCE and AC on the expression of cell protection-related proteins in RAW 264.7 cells

RAW 264.7 cells treated with DCE showed significant inductions of cell protection-related proteins, that is, HSP-27 (110%), HSP-70 (120.5%), HSP-90 (104.8%), Jun N-terminal protein kinase-1 (JNK-1, 121.3%), activating protein-1 (AP-1, 116.6%), specificity protein 1 (SP-1, 117.5%), and protein kinase C (PKC, 109.2%). The expression levels of HSP-70, JNK-1, and PKC were obviously up-regulated after DCE-2.5 treatment, and those of SP-1 and AP-1 were also obviously up-regulated after DCE-5 treatment; however, these up-regulations were dramatically reduced after DCE-10 treatment. The expression of cellular survival and membrane protection, that is, PKC, TERT, pAKT1/2/3, Muc1, and Muc4, were increased to 109.2%, 108.6%, 105.3%, 108.1%, and 111% by DCE-5 or DCE-10, respectively, whereas the expression of phosphorylated PKC (p-PKCα) showed a minimum change of less than ±5%. Alternately, the expression of p38, PTEN, and phospholipase C beta 2 (PLC-β2) changed minimally similarly to the control housekeeping proteins (Fig. 5C1). RAW 264.7 cells treated with AC-2.5 showed a slight up-regulation of AP-1 (109.6%), whereas cells treated with AC-5 showed a slight down-regulation of pAKT1/2/3 (88.7%), PKC (89.4%), PTEN (89.4%), and p38 (96.7%). AC produced non-significant changes in the expressions of other cell protection-related proteins (Fig. 5C2). These results indicated that DCE induced cell protection-related proteins in dose-dependent manner, i.e., typically up-regulated after DCE 2.5 and DCE-5 treatments but down-regulated after DCE-10 treatment, while these cell-protective effects were not observed after AC treatment.

### Effects of DCE and AC on the expression of osteogenesis-related proteins in RAW 264.7 cells

RAW 264.7 cells treated with DCE showed slight increases in the expression of osteoprotegerin (OPG, 105.4%), osteonection (105.9%), osteopontin (105.4%), osterix (108.4%), and alkaline phosphatase (ALP, 109.7%) compared with non-treated controls. The expression levels of receptor activator of nuclear factor kappa-B ligand (RANKL) and cathepsin K were obviously up-regulated to 107.7% and 106% after DCE treatment, but those of osteocalcin and HSP-90 showed a minimal change similarly to the control housekeeping proteins (Fig. 5D1). RAW 264.7 cells treated with AC showed consistent up-regulation of osteogenic proteins, that is, ALP (113.9%), RUNX2 (111.4%), osterix (106.7%), HSP-90 (106.1%), osteonectin (105.1%), osteocalcin (105.9%), and RANKL (116.8%), whereas cells treated with AC showed non-significant changes in the expression of OPG and osteopontin (Fig. 5D2). These results indicated that DCE slightly up-regulated osteogenesis-related proteins to a degree in RAW 264.7 cells compared with the non-treated control, but the expression levels were lower than those observed in response to AC treatment. However, it can be assumed that RAW 264.7 cells, which have the potential to become osteoclasts, showed a slight osteogenic effect after DCE treatment compared with the non-treated control and that RAW 264.7 cells treated with DCE might play a role in the osteogenic effect, even though they had a reduced osteogenic effect than the cells treated with AC.

### Effects of DCE on the expression of angiogenesis-related proteins in RAW 264.7 cells

**Figure 6:**
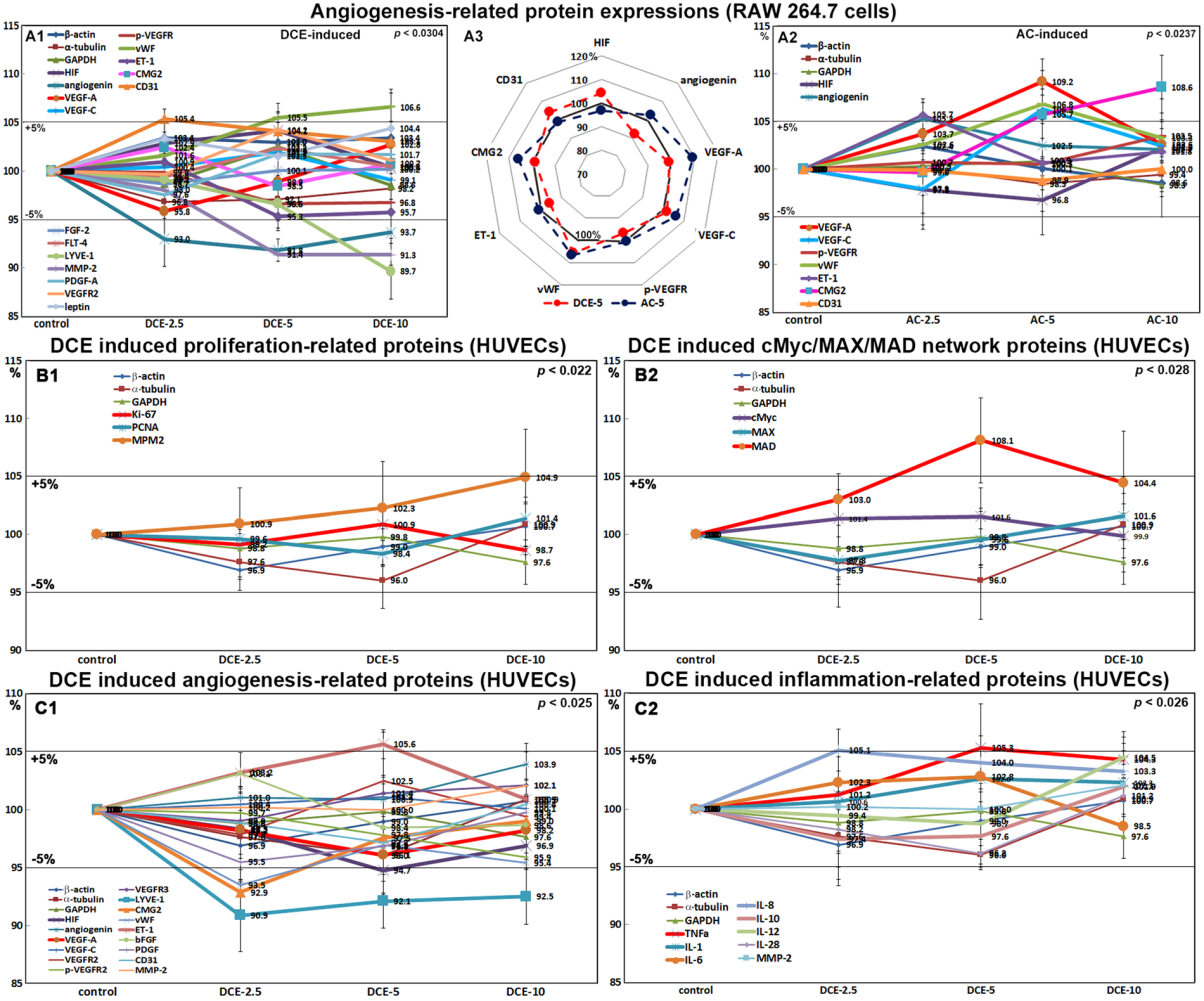
Expression of angiogenesis-related proteins (**A**) after DCE (A1) or AC (A2) treatment in RAW 264.7 cells. A3: A radial graph disclosing great contrast in protein expression between DCE-5 and AC-5 treatments. Expression of proliferation-related proteins (B1), cMyc/MAX/MAD signaling proteins (B2), angiogenesis-related proteins (C1), and inflammation-related proteins (C2) after DCE treatment in HUVECs as determined by IP-HPLC.

RAW 264.7 cells treated with DCE-2.5, -5, or -10 showed slight decreases in the expression of angiogenesis-related proteins, that is, vascular endothelial growth factor-A (VEGF-A, 95.8%), phosphorylated vascular endothelial growth factor receptor (p-VEGFR2, 96.6%), angiogenin (91.8%), lymphatic vessel endothelial hyaluronan receptor 1 (LYVE-1, 89.7%), and MMP-2 (91.3%) versus non-treated controls. However, the expression levels of vascular constriction factor and endothelin-1 (ET-1) gradually diminished to 95.7% after DCE treatment, while those of von Willebrand factor (vWF), CD31, and hypoxia-inducible factor increased slightly to 106.6%, 105.4%, and 104.2%, respectively, after DCE treatment. Alternately, the expression levels of VEGF-C, platelet-derived growth factor (PDGF), FGF-2, capillary morphogenesis protein 2 (CMG2) and vascular endothelial growth factor receptor 3 precursor (FLT4) changed less than ±5% similarly to the control housekeeping proteins (Fig. 6A1). By contrast, RAW 264.7 cells treated with AC-2.5 consistently showed a slight up-regulation of VEGF-A (109.2%), VEGF-C (106.3%), CMG2 (108.6%), angiogenin (105.3%), vWF (106.8%), and ET-1 (105.7%) (Fig. 6A2). These findings revealed weak anti-angiogenic properties of DCE by contrast to slight angiogenic properties of AC in RAW 264.7 cells.

### Effects of DCE on the expression of proliferation-related proteins in HUVECs

HUVECs treated with DCE-2.5, -5, or -10 showed almost no change in the expression of proliferation-related proteins, that is, Ki-67, proliferating cell nuclear antigen (PCNA), and MPM2, compared with non-treated controls. These expression changes were usually within ±5% similarly to the expression changes exhibited by control housekeeping proteins (Fig. 6B1). Therefore, we concluded that DCE treatment probably had no proliferative effect on HUVECs but a marked proliferative effect on RAW 264.7 cells (Fig. 2A1).

### Effects of DCE on the expression of cMyc/MAX/MAD signaling proteins in HUVECs

HUVECs treated with DCE-2.5, -5, or -10 showed almost no change in the expression of components of the cMyc/MAX/MAD network versus non-treated controls. The expression changes exhibited by cMyc, MAX, and MAD were usually within ±5% and were similar to those of control housekeeping proteins, although DCE-5 induced slight increases in MAD expression to 108.1% (Fig. 6B2). We concluded that DCE treatment did not activate the cMyc/MAX/MAD network in HUVECs, which was By contrast to the marked activation of the cMyc/MAX/MAD network in RAW 264.7 cells (Fig. 2B1).

### Effects of DCE on the expression of angiogenesis-related proteins in HUVECs

HUVECs treated with DCE-2.5, -5, or -10 showed slight decreases in the expression levels of angiogenesis-related proteins, that is, vascular endothelial growth factor-A (VEGF-A, 96%), vascular endothelial growth factor receptor 2 (VEGFR2, 97.9%), p-VEGFR2 (96.7%), von Willebrand factor (vWF, 93.5%), and platelet-derived growth factor (PDGF, 95.5%), versus non-treated controls. However, the expression levels of hypoxia inducible factor (HIF, 94.7%), lymphatic vessel endothelial hyaluronan receptor 1 (LYVE-1, 90.9%), and capillary morphogenesis protein 2 (CMG2, 92.9%) were consistently diminished after DCE-2.5 treatment, while those of vascular constriction factor and endothelin-1 (ET-1) were slightly increased to 105.6% after DCE-5 treatment. Alternately, the expression levels of angiogenin, VEGF-C, VEGFR3, bFGF, CD31, and MMP-2 changed less than ±5% similarly to the control housekeeping proteins (Fig. 6C1). These findings concur with reports on the anti-angiogenic properties of coffee-specific diterpenes, such as, cafestol and kahweol^24–27^. However, in the present study, DCE exhibited only a weak anti-angiogenic effect on HUVECs at all three doses examined.

### Effects of DCE and AC on the expressions of inflammation-related proteins in HUVECs

HUVECs treated with DCE-2.5, -5, or -10 showed almost non-significant change at less than ±5% in the expression of inflammation-related proteins, including TNFα, IL-1, IL-6, IL-8, IL-10, IL-12, IL-28, and MMP-2, versus non-treated controls. These expression changes were usually similar to those observed for control housekeeping proteins (Fig. 6C2). Therefore, we concluded that DCE likely had no inflammatory effect on HUVECs, but it induced marked anti-inflammatory effects on RAW 264.7 cells, a murine antigen presenting cell lineage (Fig. 4B1).

## Discussion

The protective and antioxidant effects of DCE in RAW 264.7 cells observed in the present study were similar to those reported for kahweol in SH-SY5Y, which up-regulated HO-1 and p38 levels^28,29^. It has been suggested that the anti-apoptosis effect of DCE in RAW 264.7 cells might play a role in the radiation-protective effect of caffeine by down-regulating BAX protein^30^. In other studies, the anti-inflammatory effects of DCE-2.5 and -5 were found to be closely related to the down-regulation of NFkB signaling^4,31^. Furthermore, the coffee-specific diterpene, kahweol, was found to suppress proliferation and induce apoptosis of human colorectal cancer cells and head and neck squamous cell carcinoma cells^32–34^. However, in the present study, DCE induced the proliferation of RAW 264.7 cells and simultaneously induced cellular immunity but reduced oncogenic protein expression, but it did not induce cellular proliferation or inflammatory reactions in HUVECs. In particular, DCE reduced the expressions of matrix inflammatory proteins (IL-6, IL-28, COX2, MMP-2, MMP-3, LTA4H, and CXCR4) and NFkB signaling proteins (p38, ERK-1, MDR, NRF2, and JNK1) independently of TNFα and NFkB expression. Therefore, it is presumed that in addition to the coffee-specific diterpene, DCE might have additional unidentified chemical elements and promote the expression of proliferative, protective, antioxidant, anti-apoptotic, innate immunity-related, and anti-inflammatory proteins in RAW 264.7 cells and thus have a net anti-carcinogenic effect.

The anti-inflammatory effects of DCE observed in RAW 264.7 cells was consistent with its strong antioxidant effect, its inactivation of NFkB signaling, and its promotion of anti-oncogenic signaling, whereas the observed increased proliferation of RAW 264.7 cells might have been due to a series of molecular signaling changes, such as increased cMyc/MAX signaling, Rb/E2F signaling, and histone activation by epigenetic modification. In particular, the marked down-regulation of p53-mediated apoptosis by DCE matched the increased expression of cellular protection-related proteins and antioxidant-related proteins, and the slight down-regulation of protein translation by DCE also matched the decreased expression of NFkB signaling-related proteins to reduce endoplasmic reticulum stress^35^. The consistent down-regulation of LTA4H and COX2 directly indicated a strong anti-inflammatory effect of DCE during the lack of COX1 expression change by DCE, and it was also remarkable that DCE induced both anti-inflammatory effect and cellular immunity-stimulating effects simultaneously^36^, which has been rarely observed for other anti-inflammatory agents.

Tea and coffee have been associated, both positively and negatively, with the risk of cardiovascular disease (CVD). Controversy still exists regarding the effects of coffee, for which there have been concerns regarding associations with hypercholesterolemia, hypertension and myocardial infarction^37^. Caffeine and kahweol are known anti-angiogenic compounds^24,27^ that may function as antitumor and anti-myocardial infarct agents. The present study showed a mild anti-angiogenesis effect after DCE treatment in RAW 264.7 cells via the down-regulation of angiogenin, VEGF-A, p-VEGFR, LYVE-1, ET-1, and MMP-2. HUVECs also showed a marked anti-angiogenic effect after DCE treatment via the down-regulation of HIF, VEGF-A, LYVE-1, CMG2, and vWF. It has also been reported that higher coffee consumption is associated with a small but significant reduction in the number of teeth with periodontal bone loss. Thereby, coffee consumption may be protective against periodontal bone loss in adult males^38^. However, many controversies have arisen due to the negative or positive osteogenic effects of coffee elements^39–41^. The present data revealed a significant osteogenic effect after DCE treatment, which was slightly lower than that observed for AC treatment. Therefore, DCE may have consistent osteogenic potential in RAW 264.7 cells.

These wide-ranging interactions between molecules in DCE and different essential signaling proteins in RAW 264.7 cells suggest that DCE molecules derived from the coffee bean matrix are bio-inert and engulfed by cells and may act to preserve and support the functions of essential proteins in RAW 264.7 cells rather than function as specific antagonists or inhibitors. In particular, DCE usually induces different beneficial effects on both RAW 264.7 cells and HUVECs in the absence of cellular stress and apoptosis, By contrast to AC. Therefore, some DCE elements other than AC elements play are considered to play an important role as chemical chaperones to assist the functions of different signaling proteins in cells^42^ and affect global protein expression in RAW 264.7 cells and HUVECs.

The non-significant changes in FAS-mediated apoptosis-related proteins induced by DCE might have been associated with the down-regulation of matrix inflammation observed in RAW 264.7 cells (almost no inflammatory signaling change was observed in HUVECs). DCE also reduced p53-mediated apoptosis signaling and enhanced cellular protection from free radical damage in RAW 264.7 cells, and it simultaneously induced cellular immunity and anti-inflammatory reactions in RAW 264.7 cells. However, it had no notable inflammatory or apoptotic effects on HUVECs. As IP-HPLC analysis revealed a series of different protein expression profiles with an accuracy of <5%, DCE appeared to have strong antioxidant, anti-inflammatory and immune stimulant, cellular proliferation and protective, anti-oncogenic and anti-angiogenic, and mild osteogenic effects on RAW 264.7 cells, and also anti-angiogenesis effects on HUVECs.

Coffee contains different polyphenol derivatives, consisting of mostly caffeine and chlorogenic acid, which have been investigated for their biological functions^43,44^. Many other coffee constituents have not been clearly identified and characterized to date. In the present study, we used DCE in the cell culture experiment rather than coffee constituents, such as kahweol or cafestol, and we examined the effects of DCE and AC at identical caffeine and chlorogenic acid concentrations. The IP-HPLC results for protein inductions by DCE or AC in RAW 264.7 cells showed that the two treatments had quite different effects on protein expression and that DCE induced more favorable effects than AC in RAW 264.7 cells. However, we also found that DCE-2.5 and -5 had more favorable effects on RAW 264.7 cells than DCE-10. AC consistently induced increases in the expression of osteogenesis-related proteins in RAW 264.7 cells with the potential to be osteoclast cells, whereas the expression of osteogenesis-related proteins was lower after DCE treatment but still slightly increased compared with the non-treated control. These findings suggest that DCE has a positive effect on bony tissue in the absence of osteoporosis^39,45^.

**Figure 7:**
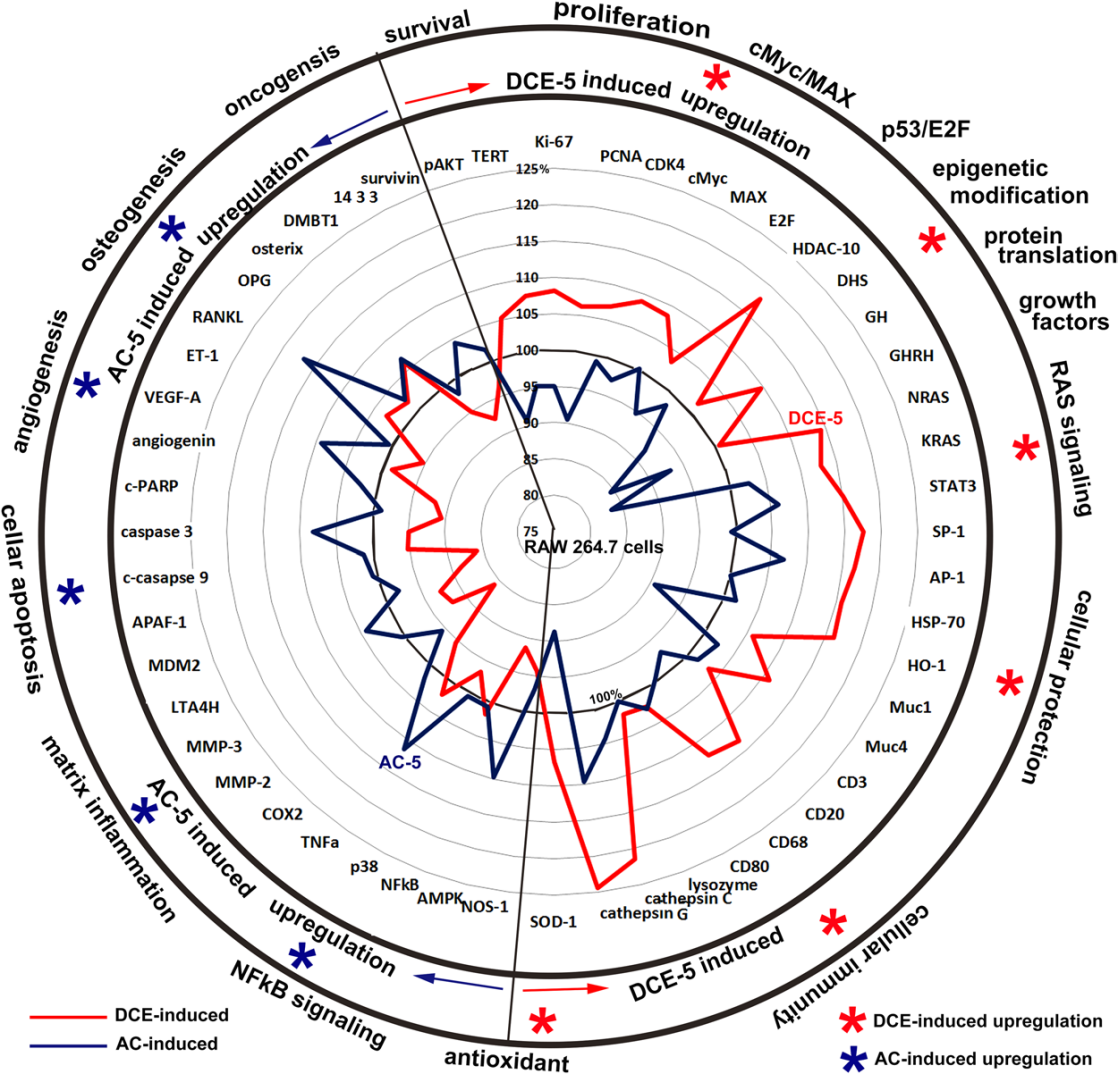
Comparison of protein expression diagrams induced by DCE-5 and AC-5 in RAW 264.7 cells. The cells showed a global circuit of molecular signaling for up-regulation and down-regulation of essential proteins to achieve different cellular functions. Red ^*^DCE-5 induced up-regulation of essential signaling proteins. Blue ^*^AC-5 induced up-regulation of essential signaling proteins.

In summary, DCE, which contains most of the minor coffee elements as well as chlorogenic acid and caffeine, induced global protein expression for essential cellular functions in RAW 264.7 cells, while AC (1 mM chlorogenic acid and 2 mM caffeine at the identical concentration to DCE) induced a very different global protein expression pattern by IP-HPLC using 180 antisera (Fig. 7). DCE induced an up-regulation of cellular proliferation, cellular protection, and antioxidant-related proteins; a down-regulations of apoptosis-related, angiogenesis-related, and oncogenic proteins; and enhanced cMyc/MAX, Rb/E2F, and RAS, growth factor signaling as well as osteogenesis in RAW 264.7 cells. The overall protein expression revealed a signaling circuit triggered by antioxidant-related proteins and genetic/epigenetic activation induced by DCE (Fig. 7, *). Therefore, it is presumed that DCE may function as strong antioxidant and genetic and epigenetic stimulant *in vitro* culture of RAW 264.7 cells.

However, these effects were somewhat muted in DCE-10-treated RAW 264.7 cells. By contrast, AC induced the expression of very distinct proteins. Our results indicated that the proteins induced by DCE would have favorable effects on RAW 264.7 cells and HUVECs, that is, DCE increased RAW 264.7 macrophage (antigen presenting cells) numbers and the expression of proteins associated positively with cellular immunity, anti-inflammatory effects, cellular protection, antioxidant effects, and anti-oncogenic effects. Furthermore, DCE slightly decreased the expression of angiogenesis-related proteins in HUVECs, which might be helpful for the treatment of cancer and cardiovascular diseases^25,27^. Our results indicated that these favorable effects of DCE in RAW 264.7 cells were probably due to unknown minor coffee elements that were not present in AC, which was prepared at caffeine and chlorogenic acid concentrations of 2 and 1 mM, respectively. Nevertheless, the current protein expression profile induced by phytochemicals, DCE and AC cannot explain most of the biological features of RAW 264.7 cells and HUVECs using the limited dosages of DEC-2.5, 5, DEC-10, AC-2.5, AC-5, and AC-10 *in vitro* cell culture. Therefore, further extensive molecular biological studies should be conducted.

## Methods

### Production of dialyzed coffee extract (DCE) and artificial coffee (AC)

First, 20 cups of coffee (20 × 150 mL = 3000 mL) were prepared from medium roasted coffee beans (*Coffea arabica* L., Nepal, roasted in Chuncheon, Korea, 20 g per a cup) by soaking them in hot water (90–95 °C) as usual for coffee drink. 300 mL aliquots of this extract were repeatedly dialyzed ten times using a permeable cellulose bag (<1000 Da; 131492, Spectra, USA) in 1500 mL double distilled water at 4 °C under stirring for 2 hours. The dialyzed coffee extract (DCE) may be concentrated with low molecular coffee elements more than the original coffee extract, and immediately preserved at −70 °C in a deep freezer until use.

**Figure 8:**
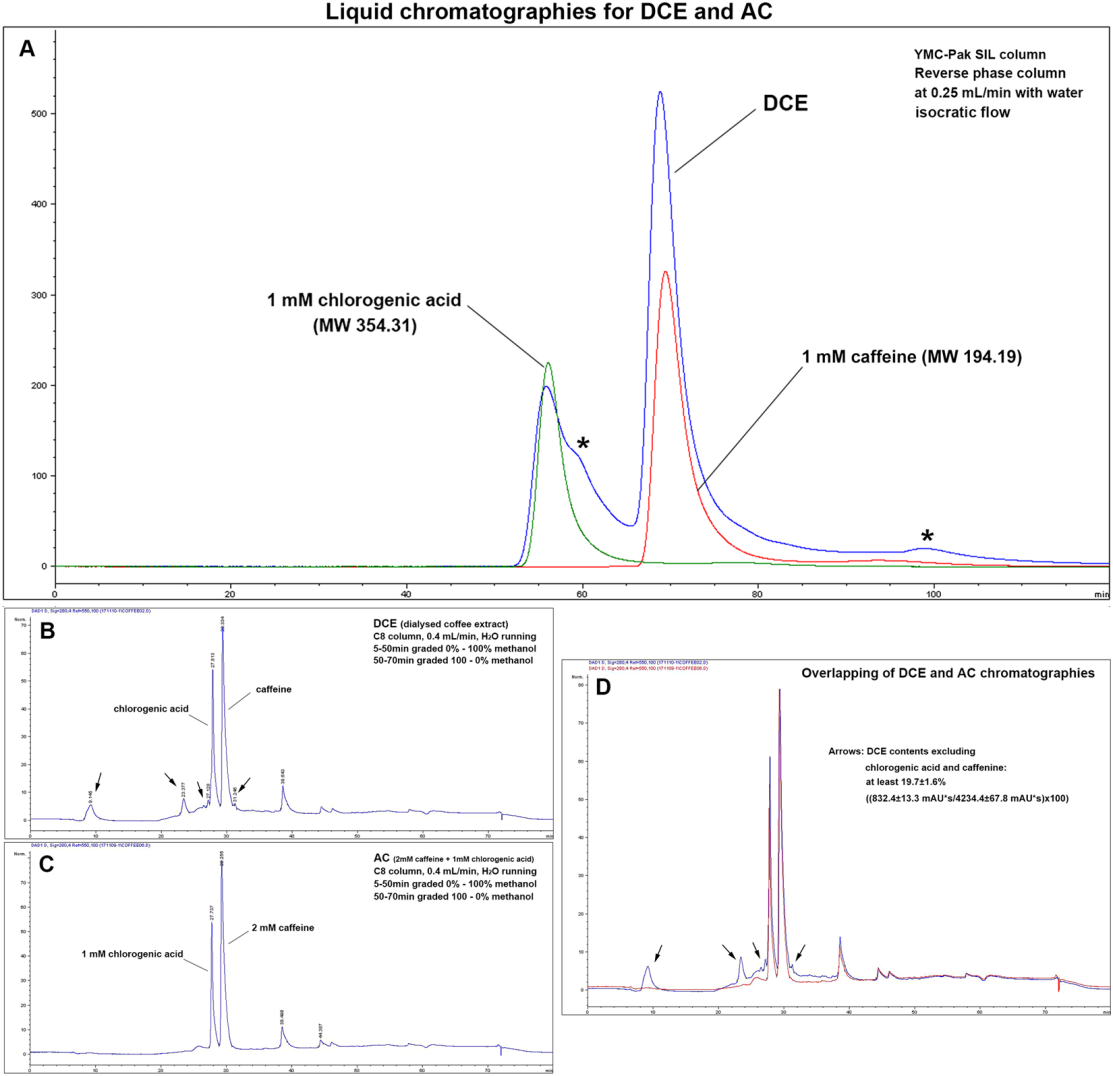
(**A**) Chromatography results for DCE (blue line), caffeine (red line), and chlorogenic acid (green line). DCE contained mostly caffeine (~2 mM, Sigma, USA), chlorogenic acid (~1 mM, Sigma, USA), and small amounts of unidentified minor coffee elements (*) in HPLC using a reverse phase silicate column (YMC-Pak). (**B**–**D)**: HPLC using C8 column, (**B**) Chromatography results for DCE, exhibiting two dominant peaks for chlorogenic acid and caffeine and several small peaks (arrows). (**C**) Chromatography results for AC, exhibiting two dominant peaks for chlorogenic acid (1 mM) and caffeine (2 mM). (**D**) Overlapping panel B and C, the two dominant peaks were almost identical in the DCE and AC graphs, and the minor small peaks (arrows) were estimated to be at least 19.7% of the total DCE contents. Five times repeated experiments were performed, and a representative chromatograph is presented.

In order to know the amount of low molecular coffee elements, non-adherent reverse phase column chromatography (YMC-Pak, Japan) at 0.25 mL/min using water as an eluent was performed using a HPLC unit (1100, Agilent, USA) and showed that the primary constituents of DCE were caffeine and chlorogenic acid. HPLC analysis of DCE revealed a caffeine concentration of ~2 mM, indicating that 150 mL DCE contained approximately 60 mg of caffeine (Fig. 8A). As 150 mL of coffee extract contained ~120 mg of caffeine, it was considered that the dialysis coefficient for minor coffee elements was approximately 50% and that 300 mL of DCE was equivalent to one cup of coffee extract (150 mL) for a human adult (mean 60 kg, 59.4 liter). Thus, 300 mL of DCE for a human adult (DCE-1) was equivalent to 0.25 mL of DCE in 50 mL of medium for RAW 264.7 cells in culture (Supplement 2).

For comparison of molecular contents between DCE and AC, the HPLC analysis using C8 column (Grace Vydac, USA) was also performed by elution with graded methanol from 0 to 100% (5–50 min) and subsequently 100 to 0% (50–70 min) in H_2_O mobile running for 80 min. The results revealed two similar peaks, chlorogenic acid and caffeine in DCE and AC, and several minor peaks that were found only in DCE (Fig. 8B–D). The total amount of these minor peaks was calculated by measuring peak areas, and then it was estimated at least 19.7 ±1.6% of the total DCE contents (Fig. 8D). In the infrared absorption using FT-IR (Spectrum Two, Perkin Elmer, USA), DCE and AC showed similar infrared absorption peaks, indicating the absence of other atypical molecules (Supplement 3). Therefore, AC consisted of 2 mM caffeine and 1 mM chlorogenic acid from chemical products (Sigma Aldrich, USA). Both DCE and AC were sterilized by filtering through 0.2-µm-pore-size membrane filter (ADVANTEC^®^, Japan), and their sterilization was confirmed by a bacterial culture test using a Lysogeny broth (LB) plate.

In order to confirm lipopolysaccharide (LPS) contamination in DCE, LPS detection assay was performed through IP-HPLC using anti-LPS antibody (Santa Cruz Biotechnology, USA). 1 mL and 2 mL DCE (experiment 1 and 2), 1 mL LPS solution (1 ng/mL, Sigma Aldrich, USA, positive control), and 1 mL distilled water (negative control) were separately analyzed by the same procedures of IP-HPLC. The peak areas of experiment 1 and 2 were similar to that of negative control, while the peak area of positive control, LPS solution, was predominantly increased (Supplement 4). These results may indicate that DCE is almost free from LPS contamination.

### RAW264.7 cell culture in the presence of DCE or AC

RAW 264.7 cells (an immortalized murine macrophage cell line; ATCC, USA) were cultured in Dulbecco’s Modified Eagle’s Medium (WelGene, Inc. Korea) supplemented with 10% (vol/vol) heat-inactivated fetal bovine serum (WelGene, Inc. Korea), 100 unit/mL penicillin, 100 μg/mL streptomycin, and 250 ng/mL amphotericin B (WelGene, Inc. Korea) in 5% CO_2_ at 37.5 °C. Cells were not stimulated with bacterial antigen (LPS) to detect native protein expressions induced by the coffee extract.

RAW 264.7 cells were separately treated with different doses of dialyzed coffee extract (DCE) equivalent to 2.5, 5, or 10 cups of coffee (DCE-2.5, DCE-5, and DCE-10, respectively); control cells were treated with 1 mL of normal saline. Cells were incubated for 12 hours, harvested with protein lysis buffer (PRO-PREP^TM^, iNtRON Biotechnology, INC, Korea), and immediately preserved at −70 °C in a deep freezer until required.

### HUVEC culture in the presence of DCE

Human umbilical cord vein endothelial cells (HUVECs; Lonza, Walkersville, MD USA) were purchased and cultured in an endothelial basal medium supplemented with 1 µg/mL hydrocortisone, 12 µg/mL bovine brain extract, 50 µg/mL gentamicin, 50 ng/mL amphotericin-B, 10 ng/mL epidermal growth factor (EGF), vascular endothelial growth factor (VEGF), basic human fibroblast growth factor (B-hFGF, FGF-2), heparin, ascorbic acid, and 10% fetal calf serum (EGM^TM^-2, Clonetics^®^, Lonza, Walkersville, MD USA) in 5% CO_2_ at 37.5 °C. Cells were tested for mycoplasma on a regular basis to ensure that only mycoplasma-free cells were assayed.

During HUVEC active growth, the experimental groups were treated with different doses of DCE equivalent to 2.5, 5, or 10 cups of coffee (DCE-2.5, DCE-5, and DCE-10, respectively); control cells were treated with 1 mL of normal saline. Cells were incubated for 12 hours, harvested with protein lysis buffer (PRO-PREP^TM^, iNtRON Biotechnology, INC, Korea), and immediately preserved at −70 °C in a deep freezer until use.

### Immunoprecipitation high performance liquid chromatography (IP-HPLC)

Approximately 100 μg of protein extract was subjected to immunoprecipitation using a protein A/G agarose column (Amicogen, Korea). Protein A/G agarose columns were separately pre-incubated with 1 μg of 189 different antisera, including growth and proliferation-related proteins (n = 9), cMyc/MAX/MAD signaling proteins (n = 3), p53/Rb/E2F signaling proteins (n = 6), epigenetic modification-related proteins (n = 7), protein translation-related proteins (n = 5), RAS signaling proteins (n = 13), growth factor-related proteins (n = 14), immunity-related proteins (n = 16), inflammation-related proteins (n = 19), NFkB signaling proteins (n = 13), cell protection-related proteins (n = 19), antioxidant-related proteins (n = 6), p53-mediated apoptosis proteins (n = 13), FAS-mediated apoptosis proteins (n = 10), oncogenic proteins (n = 7), angiogenesis-related proteins (n = 17), osteogenesis-related proteins (n = 9), and cytoplasmic housekeeping proteins (n = 3) (Supplement 5). All the antibody were commercially purchased, which were applicable to IP method and specific to the proteins of both human and mouse origin (The data sheet of antibody list was presented in Supplement 6).

Briefly, protein samples were mixed with 5 mL of binding buffer (150 mM NaCl, 10 mM Tris-HCl pH 7.4, 1 mM EDTA, 1 mM EGTA, 0.2 mM sodium vanadate, 0.2 mM PMSF and 0.5% NP-40) and incubated in protein A/G agarose columns at 4 °C for 1 hour (columns were placed on a rotating stirrer during the incubation). After washing each column with sufficient phosphate-buffered saline solution, target proteins were eluted with 150 μL of IgG elution buffer (Pierce, USA). Immunoprecipitated proteins were analyzed using a HPLC unit (1100 series, Agilent, USA) equipped with a reverse phase column and a micro-analytical detector system (SG Highteco, Korea). Elution was performed using 0.15 M NaCl, 20% acetonitrile solution at 0.4 mL/min for 30 min and detection by UV spectroscopy at 280 nm. Control and experimental samples were run sequentially to allow comparisons. For IP-HPLC, whole protein peak areas (mAU*s) were calculated by subtracting antibody peak areas of negative controls, and protein peak area square roots were calculated for normalization to the relative level of the molecular concentration (Supplement 7).

When the IP-HPLC results were compared with the western blot data of cytoplasmic housekeeping protein (β-actin), the former exhibiting minute error ranges less than ±5% could be analyzed statistically, while the latter showed a large error range of more than 20%, and thus it was almost impossible to analyze them statistically (Supplement 8). We also performed several western blot experiments using IL-10, CD20, NRAS, etc., and compared with the results of IP-HPLC. Western blot results showed relatively irregular protein expression changes depending on the increase of DCE dose, i.e., DCE-0, DCE-2.5, DCE-5, and DCE-10, compared to the IP-HPLC results. Although western blot data were plotted similar trends of protein expression to IP-HPLC data, the protein expression changes of western blot data were not proportional and showed a feature of fluctuation in line graphs compared to those of IP-HPLC data (Supplement 9). Particularly, repeated experiment of IP-HPLC for each protein expression, 4–10 times, produced accurate IP efficiency with minimum error range (less than ±5%) (The raw data sheets of IP-HPLC analysis were presented in Supplements 10–12). Therefore, in the present study we preferred to do IP-HPLC analysis rather than western blot analysis in order to analyze the protein expression changes statistically.

### Statistical analysis

Proportions (%) of experimental and control groups were plotted (Supplement 4), and the analysis was repeated two to six times (until the mean and standard deviations were ≤±5%). The results were analyzed using the Chi-squared test^21,46,47^. The expression of control housekeeping proteins, that is, *β*-actin, *α*-tubulin, and glyceraldehyde 3-phosphate dehydrogenase (GAPDH), were relatively unchanged (≤5%) by DCE-2.5, 5, or 10. Protein expression changes were defined as non-significant for changes ≤±5%, slight for ±5–10%, marked for ±10–20%, and great for ≥±20%.

## Electronic supplementary material

dataset 1

dataset 2

dataset 3

dataset 4

dataset 5
