# Supplementary material for: In vitro protein expression changes in RAW 264.7 cells and HUVECs treated with dialyzed coffee extract by immunoprecipitation high performance liquid chromatography
Source: Sci Rep. 2018 Sep 14;8:13841. doi: 10.1038/s41598-018-32014-z (PMC6138699; doi:10.1038/s41598-018-32014-z)
Supplement: Supplementary file 1 — dataset 1 [file 41598_2018_32014_MOESM1_ESM.doc]

***In vitro* protein expression changes in RAW 264.7 cells and HUVECs treated with dialyzed coffee extract by immunoprecipitation high performance liquid chromatography**

Cheol Soo Yoon^1^, Min Keun Kim^2^, Yeon Sook Kim^3^, Suk Keun Lee^1^*

^1^Department of Oral Pathology, ^2^Department of Oral and Maxillofacial Surgery, College of Dentistry, Gangneung-Wonju National University, and Institute of Oral Science, Gangneung; ^3^Department of Dental Hygiene, College of Health Sciences, Cheongju University, Cheongju, Korea

Supplement 1.

Table 1. Statistical analysis data for cell counting after DCE and AC treatment

| Cell counting after treatments of DCE and AC | | | | | | | | | |  |
| --- | --- | --- | --- | --- | --- | --- | --- | --- | --- | --- |
| No. | **DCE-cont** | **DCE-2.5** | **DCE-5** | **DCE-10** |  | **AC-cont** | **AC-2.5** | **AC-5** | **AC-10** |  |
| 1 | 359 | 362 | 394 | 430 |  | 335 | 334 | 352 | 207 |  |
| 2 | 319 | 363 | 426 | 408 |  | 333 | 270 | 292 | 228 |  |
| 3 | 299 | 392 | 450 | 365 |  | 330 | 238 | 304 | 237 |  |
| 4 | 325 | 325 | 445 | 387 |  | 372 | 307 | 350 | 262 |  |
| 5 | 315 | 293 | 350 | 370 |  | 287 | 271 | 281 | 279 |  |
| 6 | 337 | 324 | 400 | 316 |  | 341 | 280 | 369 | 233 |  |
| 7 | 342 | 335 | 402 | 318 |  | 323 | 224 | 313 | 268 |  |
| 8 | 329 | 311 | 365 | 343 |  | 327 | 279 | 284 | 286 |  |
| 9 | 345 | 285 | 406 | 297 |  | 301 | 312 | 301 | 263 |  |
| 10 | 296 | 297 | 353 | 390 |  | 261 | 258 | 343 | 230 |  |
| 11 | 364 | 310 | 406 | 264 |  | 328 | 334 | 323 | 196 |  |
| 12 | 327 | 373 | 360 | 278 |  | 343 | 290 | 327 | 295 |  |
| 13 | 296 | 359 | 325 | 334 |  | 296 | 348 | 295 | 248 |  |
| 14 | 344 | 357 | 356 | 255 |  | 256 | 281 | 291 | 234 |  |
| 15 | 330 | 402 | 343 | 284 |  | 299 | 362 | 287 | 247 |  |
| 16 | 355 | 382 | 299 | 322 |  | 309 | 283 | 286 | 271 |  |
| 17 | 331 | 282 | 342 | 333 |  | 324 | 295 | 251 | 271 |  |
| 18 | 321 | 335 | 306 | 431 |  | 310 | 304 | 272 | 357 |  |
| 19 | 352 | 341 | 380 | 399 |  | 327 | 356 | 300 | 280 |  |
| 20 | 315 | 350 | 370 | 391 |  | 302 | 338 | 312 | 278 |  |
| 21 | 333 | 341 | 384 | 389 |  | 328 | 344 | 320 | 245 |  |
| 22 | 321 | 328 | 370 | 357 |  | 317 | 332 | 299 | 251 |  |
| 23 | 335 | 351 | 390 | 333 |  | 299 | 305 | 297 | 247 |  |
| 24 | 341 | 328 | 362 | 326 |  | 321 | 348 | 300 | 241 |  |
| 25 | 331 |  | 370 | 351 |  |  | 349 |  | 259 |  |
| 26 |  |  | 368 | 303 |  |  | 332 |  |  |  |
| 27 |  |  | 369 | 389 |  |  | 325 |  |  |  |
| 28 |  |  |  | 321 |  |  | 356 |  |  |  |
| 29 |  |  |  | 335 |  |  | 318 |  |  |  |
| 30 |  |  |  | 432 |  |  | 391 |  |  |  |
| 31 |  |  |  | 311 |  |  | 337 |  |  |  |
| 32 |  |  |  | 389 |  |  | 337 |  |  |  |
| 33 |  |  |  | 410 |  |  | 348 |  |  |  |
| 34 |  |  |  | 420 |  |  | 295 |  |  |  |
| 35 |  |  |  |  |  |  | 296 |  |  |  |
| % |  | 1.4 | 12.1 | 5.6 |  |  | -1.4 | -3.9 | -19.7 |  |
| average | 330.5 | 338.6 | 373.7 | 352.4 |  | 315.4 | 314.1 | 306.2 | 256.5 |  |
| STD | 18.16 | 32.47 | 36.33 | 49.74 |  | 25.49 | 37.87 | 27.37 | 31.84 |  |
| *p* | 0.0030 | 0.0092 | 0.0095 | 0.0199 | 0.0104 | 0.0065 | 0.0145 | 0.0080 | 0.0154 | 0.0111 |

**Supplement 2.** Calculation of DCE dose for culture media to achieve equivalence in human adults

| **Coffee** | **Ordinary coffee extract** | **Dialyzed coffee extract (DCE)** |
| --- | --- | --- |
| caffeine concentration | 120 mg/150 mL | 60 mg/150 mL |
| dialysis coefficient |  | 50% |
| one cup for a human adult (60 Kg, 59.4 L) | 150 mL | 300 mL (DCE-1) |
| DCE-1 to 50 mL culture media |  | 0.25 mL |
| DCE-2.5 to 50 mL culture media |  | 0.625 mL |
| DCE-5 to 50 mL culture media |  | 1.25 mL |
| DCE-10 to 50 mL culture media |  | 2.5 mL |


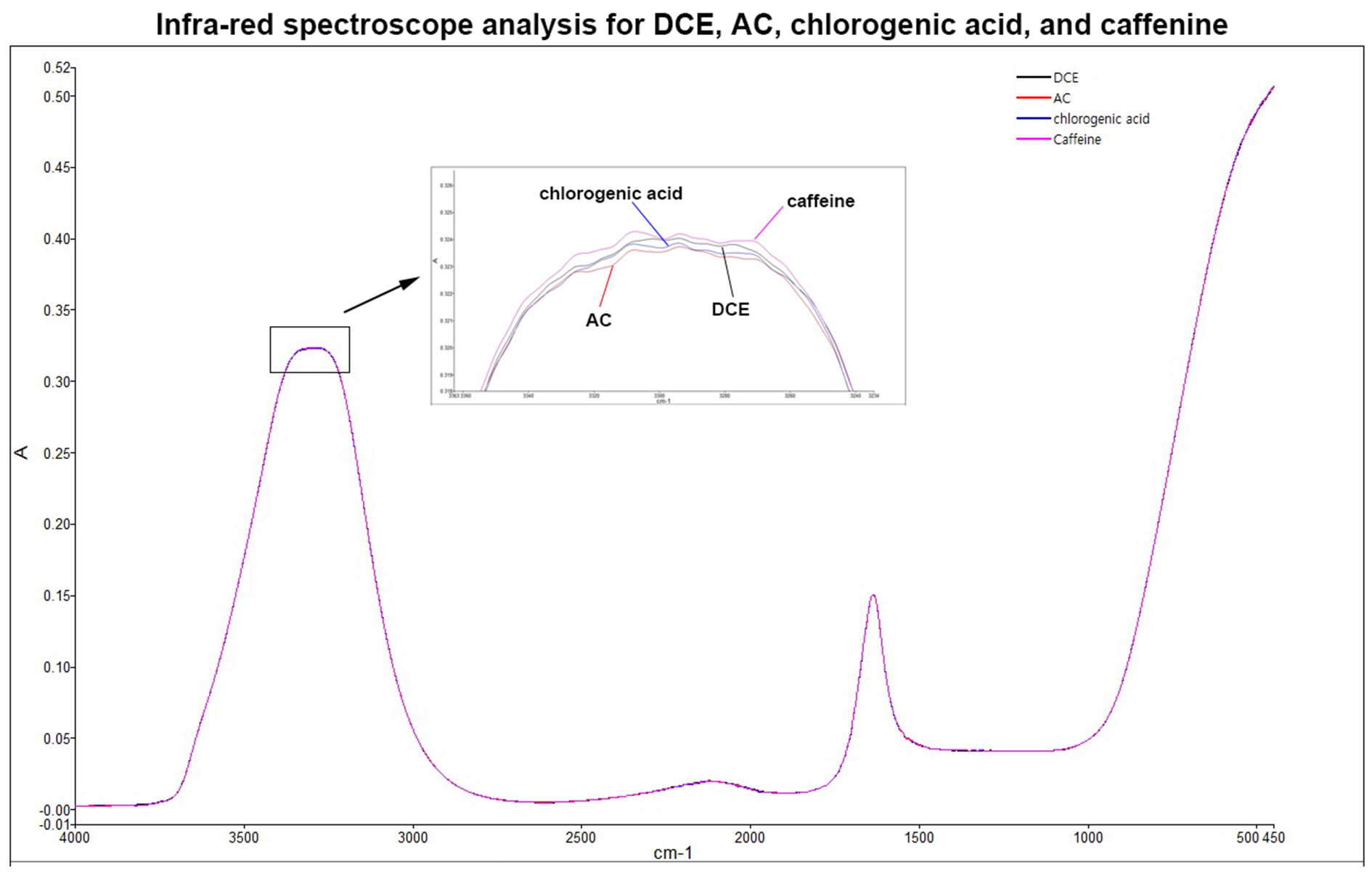


**Supplement 3.** Infra-red spectroscope analysis for DCE, AC, chlorogenic acid, and caffeine, exhibiting similar molecular interaction pattern in water solution without atypical contamination in DCE and AC used in this study.


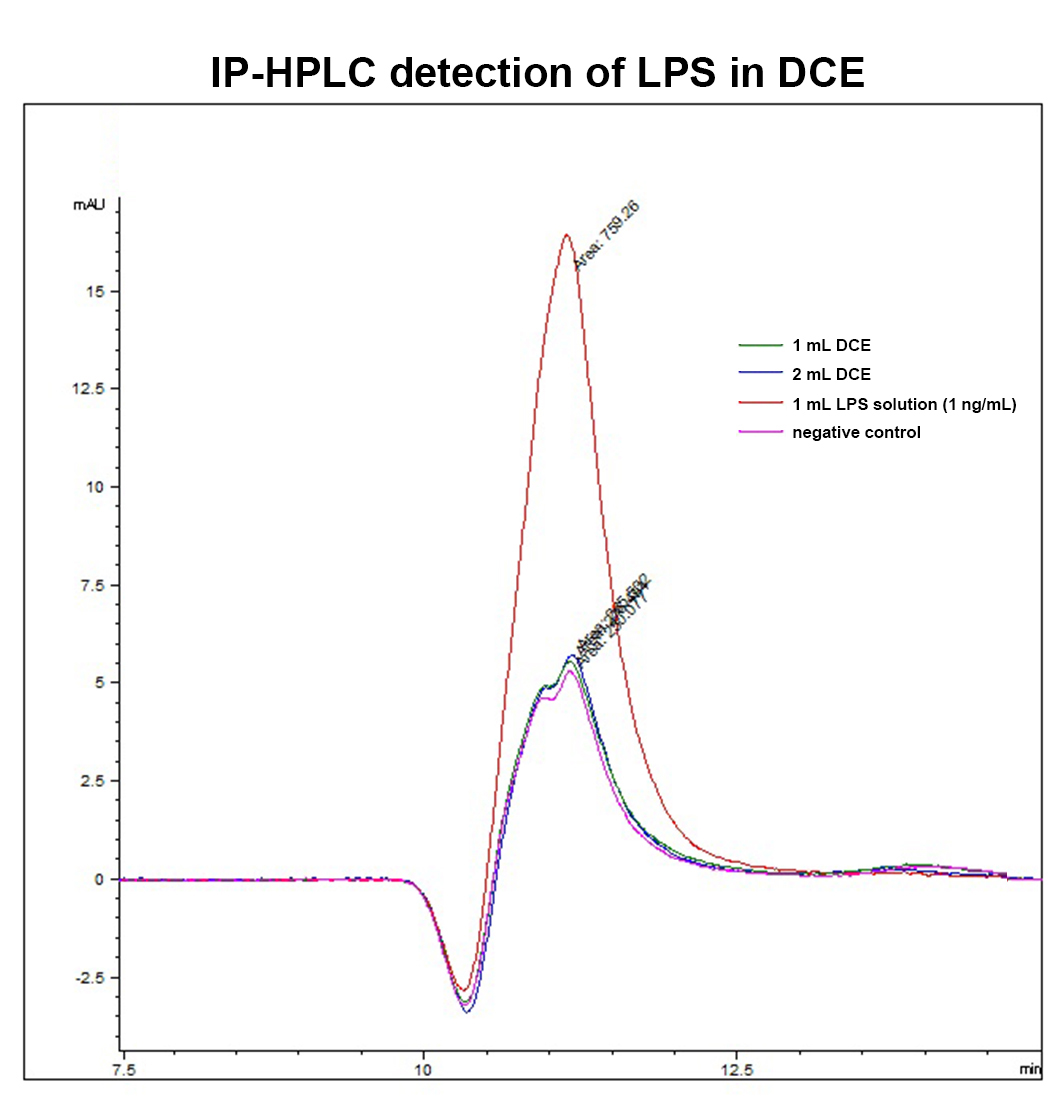


**Supplement 4.** A chromatography for LPS detection assay through IP-HPLC. Pink line: negative control using distilled water. Green line: experiment 1 to detect LPS in 1mL DCE. Blue line: experiment 2 to detect LPS in 2 mL DCE. Red line: positive control using 1 mL LPS solution (1 µg/mL). The peak areas of experiment 1 and 2 were similar to that of negative control, while the peak area of positive control, LPS solution, was predominantly increased. These results may indicate that DCE is almost free from LPS contamination.

**Supplement 5.** Antibodies used in this study.

| **Signaling proteins** | **No.** | **Antibodies** |
| --- | --- | --- |
| Cellular proliferation | 9 | Ki-67**^*^**, PCNA**^*^**, CDK4^*^, PLK4^*^, MPM2**^*^**, p14**^*^**, p16^*^, p21^*^, p27^*^ |
| cMyc/MAX/MAD signaling | 3 | cMyc^*^, MAX**^*^**, MAD**^*^** |
| p53/Rb/E2F signaling | 4 (2) | p53, Rb-1^#^, E2F-1^*^, MDM2, (CDK4, p21) |
| Epigenetic modification | 7 | DMAP1, histone H1**^*^**, KDM4D ^$^, HDAC-10^$^, MBD4, DNMT1 |
| Protein translation signaling | 5 | DOHH**^*^**, DHS**^*^**, elF5A-1^$^, elF5A-2^$^, eIF2AK3 |
| RAS signaling | 9 (4) | NRAS^$^, KRAS^$^, STAT3^*^, P13K**^*^**, JNK-1**^*^**, ERK-1**^*^**, PTEN^*^, RAF-B, PGC-1α, (pAKT1/2/3, mTOR, PKC, p-PKC) |
| Growth factor signaling | 14 | bFGF^*^, HGF**^*^**, TGF-β1^#^, SMAD4**^*^**, IGF-1**^*^**, IGFIIR**^*^**, GH**^*^**, GHRH**^*^**, HER1**^*^**, HER2**^*^**, ERβ,**^*^** FGF-1, FGF-2, Met |
| NFkB signaling | 9 (4) | NFkB**^*^**, IKK**^*^**, GADD45**^*^**, mTOR@, p38, p-p38**^*^**, NRF2, JNK1, MDR, (ERK-1, PTEN, pAKT1/2/3, TNFα) |
| Immunity signaling | 16 | CD3**^*^**, CD20**^*^**, CD28**^*^**, CD31**^*^**, CD34**^*^**, CD40**^*^**, CD54**^*^**, CD56**^*^**, CD68**^*^**, CD80**^*^,** CD99, α1-antitrypsin**^*^**, LL-37**^*^**, cathepsin C**^*^**, cathepsin G**^*^**, cathepsin K**^*^** |
| Inflammatory signaling | 19 | TNFα@, IL-1**^*^**, IL-6**^*^**, IL-8**^*^**, IL-10**^*^**, IL-12**^*^**, IL-28**^*^**, COX-1**^*^**, COX-2**^*^**, lysozyme**^*^**, M-CSF**^*^**, MMP-1**^*^**, MMP-2**^*^**, MMP-3**^*^**, MMP-9**^*^**, MMP-10**^*^,** MMP-12, LTA4H, CXCR4 |
| Cell protection | 17 (2) | HSP-27**^*^**, HSP-70**^*^**, HSP-90**^*^**, AP-1**^*^**, SP-1**^*^**, SP-3**^*^**, p38**^*^**, PKC**^*^**, pAKT1/2/3**^*^**, p63, TGase-2, caveolin, Muc1, Muc4, PLC- β2, p-PKCα, leptin, (PTEN, TERT) |
| Antioxidant-related proteins | 6 | HO-1**^*^**, SOD-1**^*^**, GST**^*^**, LC3**^*^**, AMPK**^*^**, NOS-1, |
| p53-mediated cellular apoptosis | 10 (3) | p53^*^, MDM2**^*^**, BCL2^*^, BAX^*^, BAD^*^, BAK**^*^**, AIF**^*^**, APAF-1**^*^**, caspase 9**^*^**, c-caspase 9**^*^**, (caspase 3, c-caspase 3, BID) |
| FAS-mediated cellular apoptosis | 10 | FASL**^*^**, FAS**^*^**, FADD**^*^**, FLIP**^*^**, BID**^*^**, caspase 8^*^, caspase 3^*^, c-caspase 3^*^, PARP^*^, c-PARP^*^ |
| Oncogenic proteins | 7 | CEA^$^, 14-3-3^*^, survivin@, DMBT1^*^, TERT**^*^**, BRCA-1, BRCA-2 |
| Angiogenesis-related proteins | 14 (3) | HIF^&^, VEGF-A**^*^**, VEGF-C**^*^**, VEGFR2**^*^**, p-VEGFR2**^*^**, angiogenin**^*^**, LYVE-1**^*^**, CMG2^$^, vWF^$^, ET-1**^*^**, FGF-2^&^, PDGF-A**^*^**, MMP-2**^*^**, FLT4, (CD31, MMP-2, leptin) |
| Osteogenesis-related proteins | 8 (1) | RANKL, OPG, osteonectin, osteopontin, osteocalcin, RUNX2, ALP, osterix, (cathepsin K) |
| Control cytoplasmic proteins | 3 | α-tubulin^*^, β-actin**^*^**, GAPDH**^*^** |
| Total | 170 (19) |  |

**^*^** Santa Cruz Biotechnology, USA; ^#^ DAKO, Denmark; ^$^ Neomarkers, CA, USA; @ ZYMED, CA, USA; ^&^Abcam, Cambridge, UK

**Abbreviations:** α1-AT; α-1 antitrypsin, ALP; alkaline phosphatase, AMPK; AMP-activated protein kinase, APAF-1; apoptotic protease-activating factor 1, AP-1; activating protein-1, BAD; BCL2 associated death promoter, BAK; BCL2 antagonist/killer, BAX; BCL2 associated X, BCL-2; B-cell leukemia/lymphoma-2, BID; BH3 interacting-domain death agonist, BRCA-1; Breast cancer type1 susceptibility protein, BRCA-2; Breast cancer type2 susceptibility protein, CASP 3; caspase 3, c-CASP 3; cleaved-caspase 3, CASP 8; caspase 8, CASP 9; caspase 9, c-CASP 9; cleaved-caspase 9, CD3; cluster of differentiation 3, CDK4; cyclin dependent kinase 4, CEA; carcinoembryonic antigen, cMyc; V-myc myelocytomatosis viral oncogene homolog, CMG2: capillary morphogenesis protein 2, COX-1; cyclooxygenase-2, COX-2; cyclooxygenase-2, c-PARP; cleaved- PARP(poly-ADP ribose polymerase), CXCR4; C-X-C chemokine receptor type 4, DMAP1; DNA methytransferase 1 associated protein, DMBT1; deleted in malignant brain tumors 1, DNMT1; DNA(cytosine-5)-methyltransferase1, DOHH; deoxyhypusine hydroxylase, DHS; deoxyhypusine synthase, E2F-1; transcription factor, elF5A-1; eukaryotic translation initiation factor 5A-1, elF5A-2; eukaryotic translation initiation factor 5A-2, eIF2AK3 (PERK; protein kinase R(PKR)-like endoplasmic reticulum kinase); eukaryotic translation initiation factor 2-alpha kinase 3, ERβ; estrogen receptor beta, ERK; extracellular signal-regulated protein kinases, ET-1: endothelin-1, FAS; CD95/Apo1, FASL; FAS ligand, FADD; FAS associated via death domain, FGF-1; fibroblast growth factor-1, FLIP; FLICE-like inhibitory protein, vascular endothelial growth factor receptor 3 precursor (FLT4), GADD45; growth arrest and DNA-damage-inducible 45, GAPDH; glyceraldehyde 3-phosphate dehydrogenase, GH; growth hormone, GHRH; growth hormone-releasing hormone, GST, HDAC-10, HIF: hypoxia inducible factor-1α, Histone H1, HO-1; hemoxygenase 1, HER1; human epidermal growth factor receptor 1, HGF; hepatocyte growth factor, hTERT; human telomerase reverse transcriptase, HSP-70; heat shock protein-70, HSP-90; heat shock protein-90, IKK; ikappaB kinase, IGF-1, IGFIIR, IL-1; interleukin-1, JMJD2D, JNK-1; Jun N-terminal protein kinase, KDM4D; Lysine-Specific Demethylase 4D, KRAS; V-Ki-ras2 Kirsten rat sarcoma viral oncogene homolog, LC3; microtubule-associated protein 1A/1B-light chain 3, LTA4H; Leukotriene A4 hydrolase, LYVE-1: lymphatic vessel endothelial hyaluronan receptor 1, MAX; myc-associated factor X, MBD4; methyl-CpG-binding domain protein 4, M-CSF; macrophage colony-stimulating factor, MDM2; mouse double minute 2 homolog, MPM2: mitotic protein monoclonal 2, MDR; Monoclonal Anti-P-Glycoprotein, Met; MMP-1; matrix metalloprotease-1, MMP2; matrix metalloproteinase-2, mTOR; mammalian target of rapamycin, Muc1; mucin1, cell surface associated, Muc4; mucin4, cell surface associated, NFkB; nuclear factor kappa-light-chain-enhancer of activated B cells, NOS-1; NRAS; neuroblastoma RAS Viral Oncogene homolog, NRF2; nuclear factor (erythroid-derived)-like 2, OPG; osteoprotegerin ,OPN; osteopontin, OSX; osterix, pAKT; v-akt murine thymoma viral oncogene homolog, p-Akt1/2/3 phosphorylated (p-Akt, Thr 308), PARP; poly-ADP ribose polymerase, P13K; phosphatidylinositol-3-kinases, PCNA; proliferating cell nuclear antigen, PDGF: platelet-derived growth factor, PGC-1α; Peroxisome Proliferator-Activated Receptor-γ-Coactivator1α, PKC; protein kinase C, PLC- β2; Phospholipase C β2, p-PKCα; protein kinase C alpha, p-p38; phosphor-p38, PTEN; phosphatase and tension homolog, RAF-B; Serine/threonine-specific protein kinase, Rb-1; retinoblastoma-1, RANKL; receptor activator of nuclear factor kappa-B ligand, RUNX2; Runt-related transcription factor 2, SAK; serine/threonine-protein kinase, SMAD4; mothers against decapentaplegic, drosophila homolog 4, SOD-1; superoxide dismutase-1, SP-1; specificity protein 1, SP-3; specificity protein 3, STAT3; signal transducer and activator of transcription-3, TGase-2; transglutaminase-2, TGF-β1; transforming growth factor-β1, TNF-α; tumor necrosis factor-α, β-actin, 14-3-3, VEGF vascular endothelial growth factor, VEGFR2: vascular endothelial growth factor receptor 2, p-VEGFR2: vascular endothelial growth factor receptor 2 (Y951), vWF: von Willebrand factor. The number of antibodies overlapped; ( ).


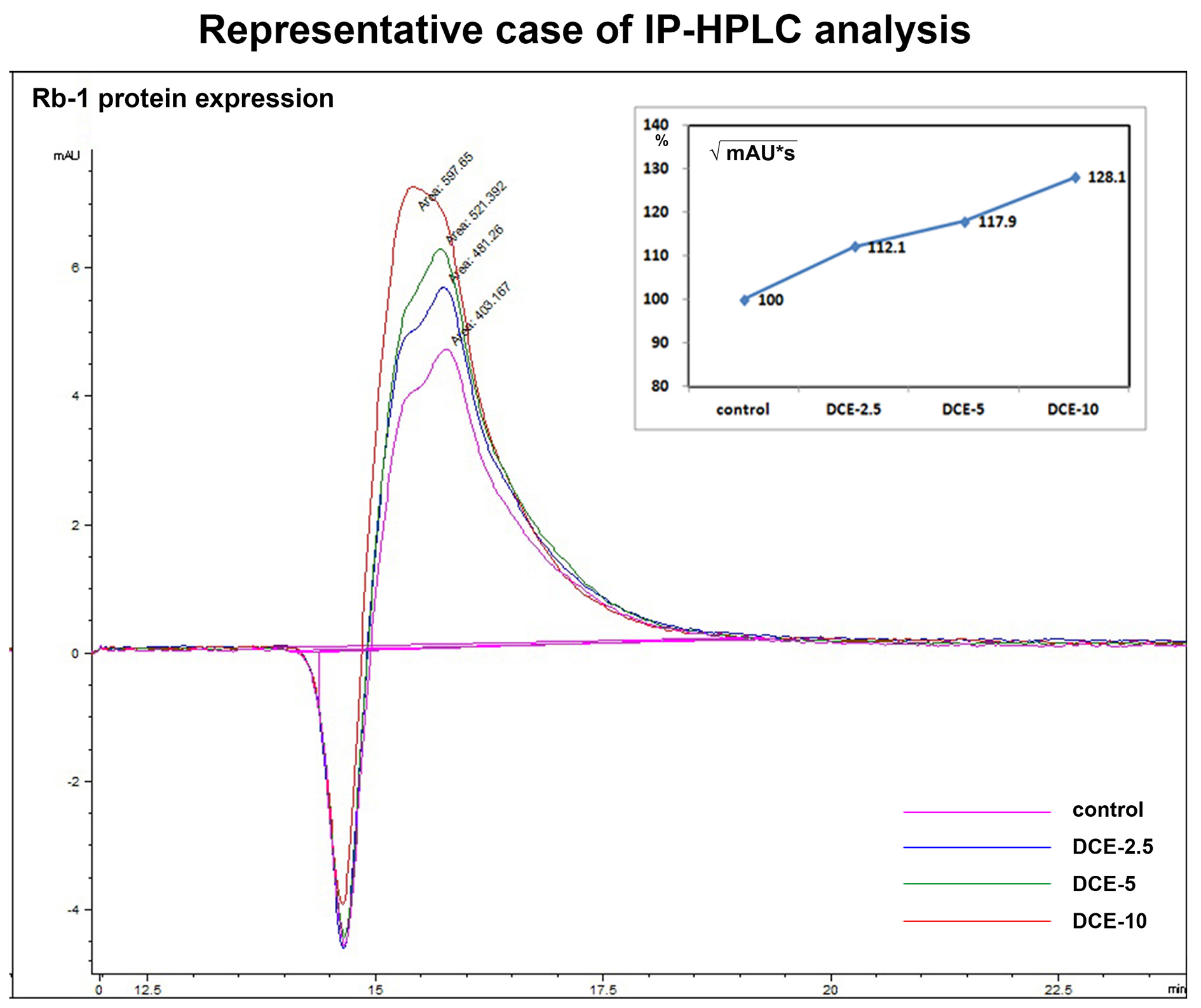


**Supplement 7.** Representative IP-HPLC chromatograms for the analysis of protein expression levels in the control and experimental groups.


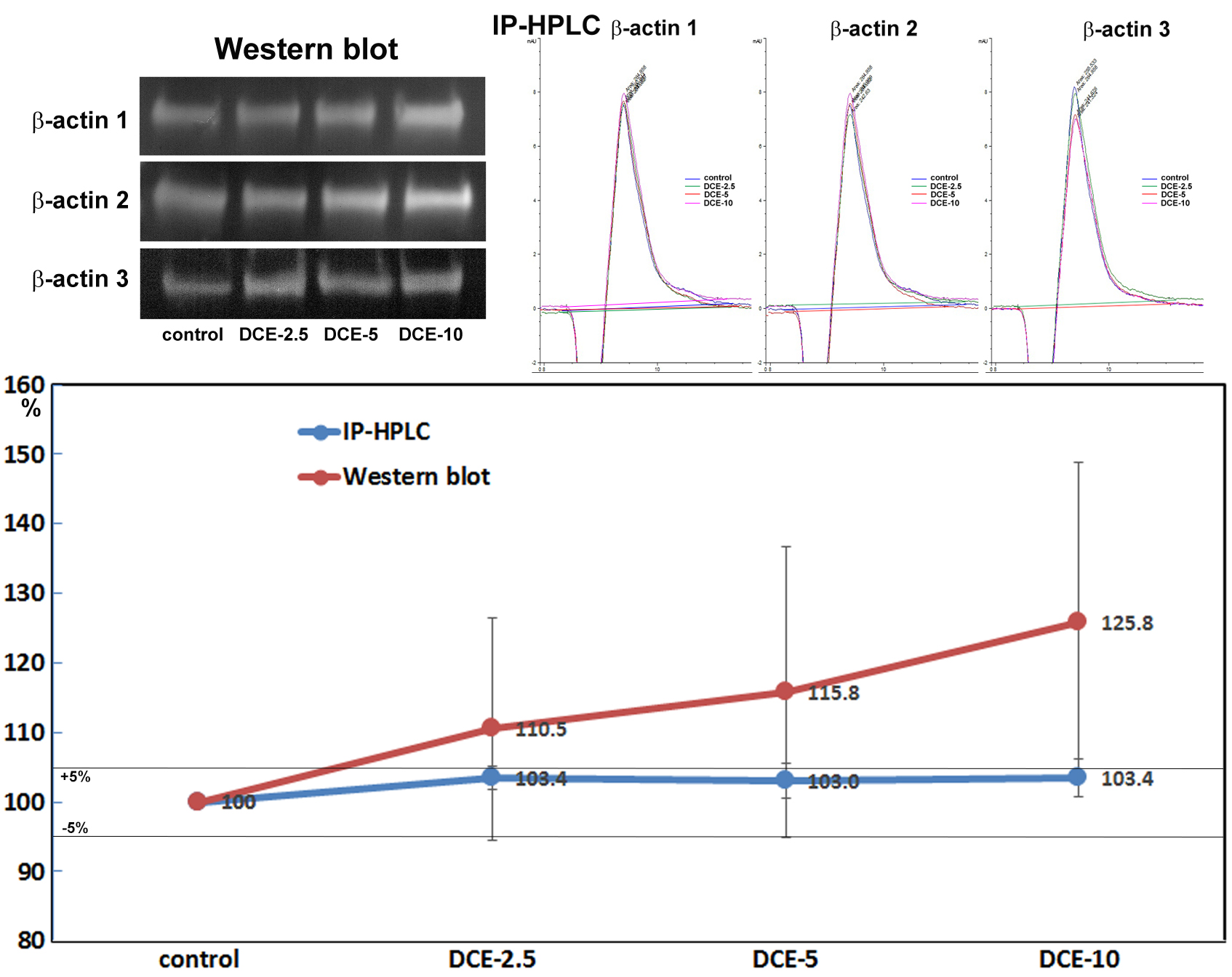


**Supplement 8.** β-Actin expression in DCE-treated RAE 264.7 cells was explored through western blot and IP-HPLC. Densitometry data of triplicated western blot (red line) showed big standard deviation (16.1 – 23.2 %), while triplicated IP-HPLC data (blue line) showed relatively small standard deviation (1.7 – 2.7%). Therefore, the latter was available to perform statistical analysis contrary to the former.


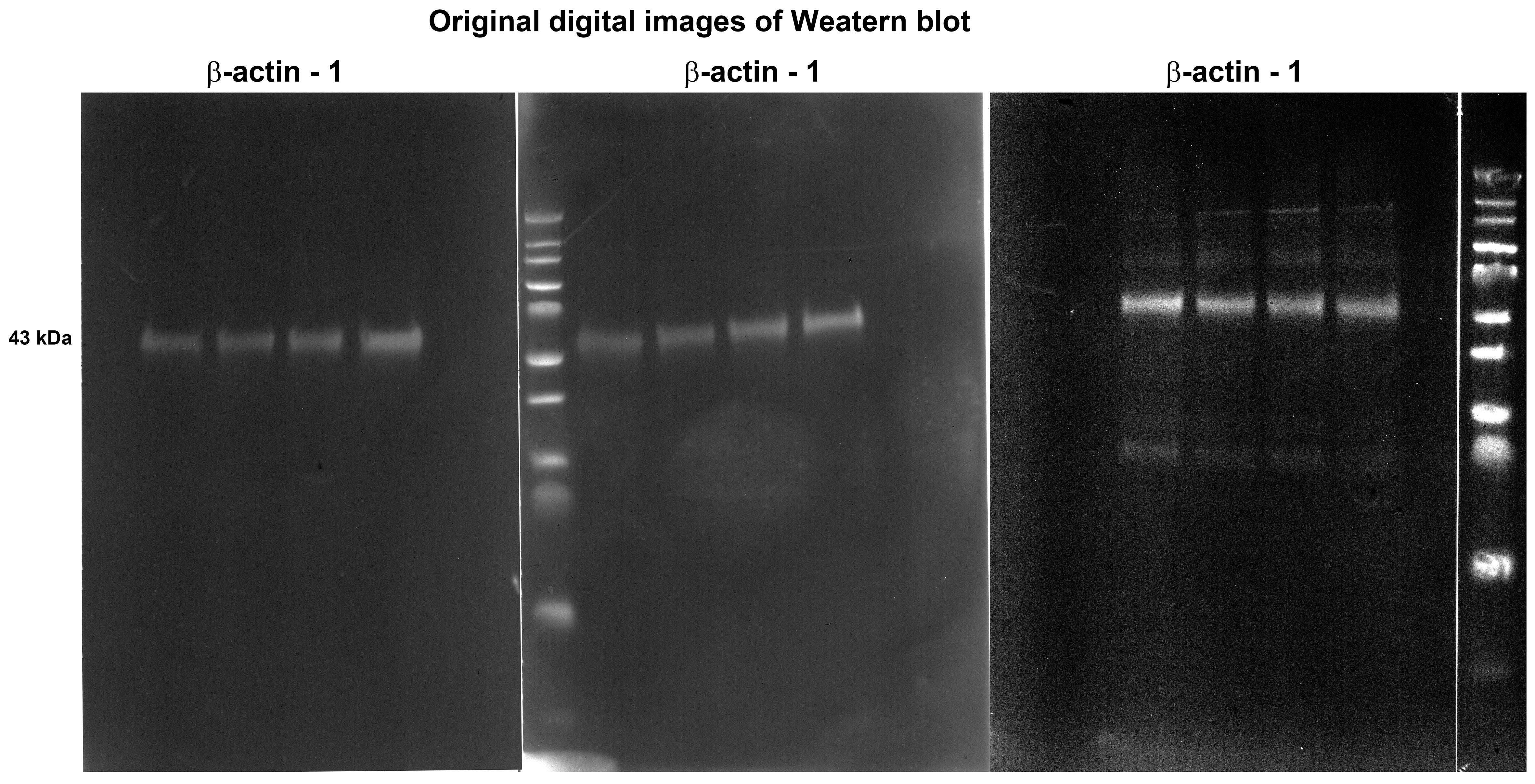


**Supplement 8-1.** Original digital Images of β-actin-1, -2, and -3 which were used in supplement 6 for the compliance with the digital images and integrity policies.


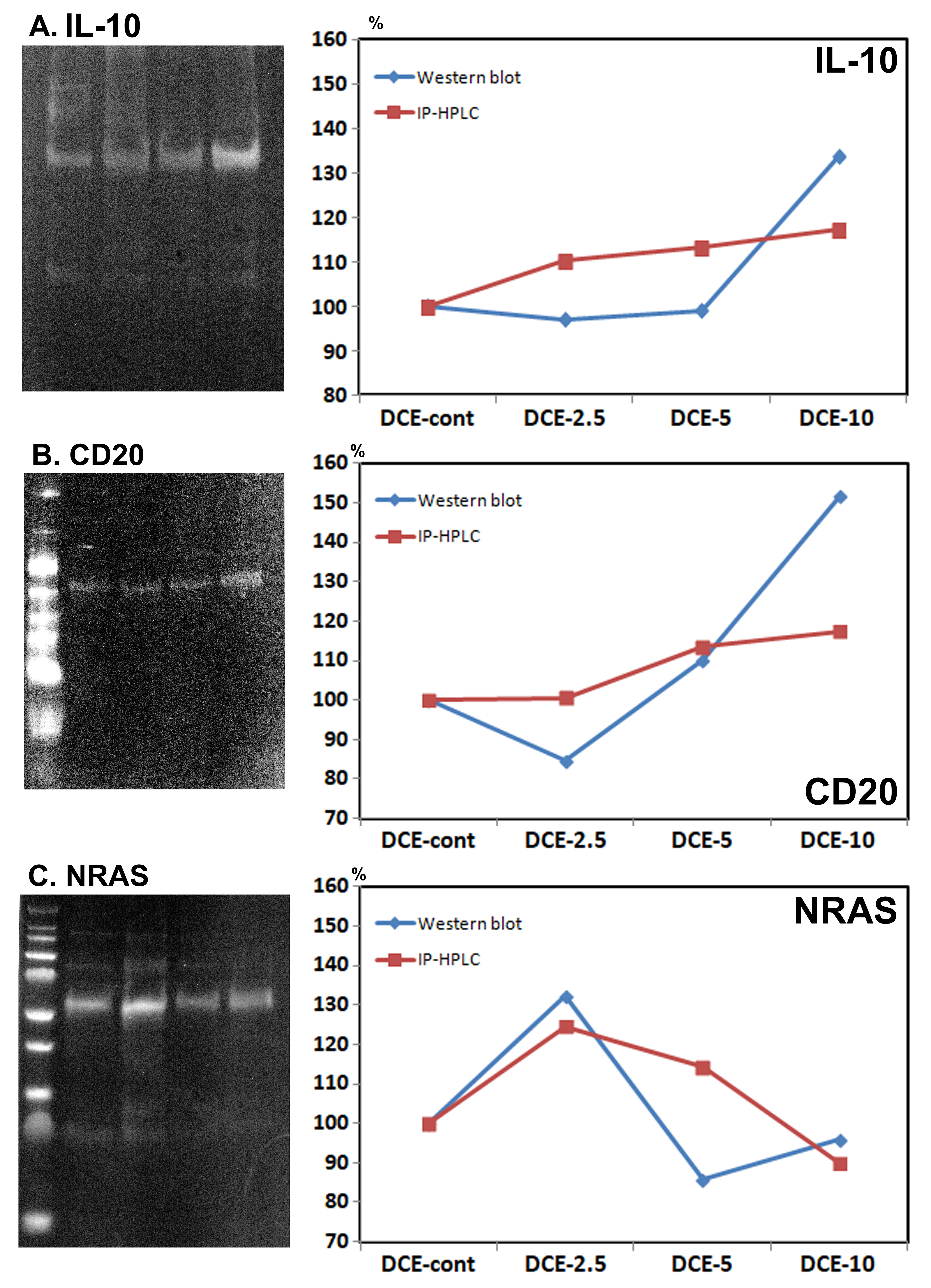


**Supplement 9.** Comparison of protein expression changes between western blot and IP-HPLC performed with same protein samples using IL-10, CD20, and NRAS antibodies. Western blot results showed relatively irregular protein expression changes depending on the increase of DCE dose, i.e., control, DCE-2.5, DCE-5, and DCE-10 compared to the IP-HPLC results. However, western blot data were plotted similar trends of protein expression to IP-HPLC data, but the protein expression changes of western blot data were not proportional and showed a feature of fluctuation in line graphs (A-C) compared to those of IP-HPLC data.
